# Functional Analysis of P450 Monooxygenase SrrO in the Biosynthesis of Butenolide-Type Signaling Molecules in *Streptomyces* *rochei*

**DOI:** 10.3390/biom10091237

**Published:** 2020-08-25

**Authors:** Aiko Teshima, Nozomi Hadae, Naoto Tsuda, Kenji Arakawa

**Affiliations:** 1 Department of Molecular Biotechnology, Graduate School of Advanced Sciences of Matter, Hiroshima University, 1-3-1 Kagamiyama, Higashi-Hiroshima, Hiroshima 739-8530, Japan; teshima-ai@hiroshima-u.ac.jp (A.T.); aaeooi2@gmail.com (N.H.); naoto.tsuda@ncx.nagase.co.jp (N.T.); 2 Unit of Biotechnology, Graduate School of Integrated Sciences for Life, Hiroshima University, 1-3-1 Kagamiyama, Higashi-Hiroshima, Hiroshima 739-8530, Japan

**Keywords:** *Streptomyces*, signaling molecule, biosynthesis, cytochrome P450, secondary metabolite

## Abstract

*Streptomyces rochei* 7434AN4 produces two structurally unrelated polyketide antibiotics lankacidin and lankamycin, and their biosynthesis is tightly controlled by butenolide-type signaling molecules SRB1 and SRB2. SRBs are synthesized by SRB synthase SrrX, and induce lankacidin and lankamycin production at 40 nM concentration. We here investigated the role of a P450 monooxygenase gene *srrO* (*orf84*), which is located adjacent to *srrX* (*orf85*), in SRB biosynthesis. An *srrO* mutant KA54 accumulated lankacidin and lankamycin at a normal level when compared with the parent strain. To elucidate the chemical structures of the signaling molecules accumulated in KA54 (termed as KA54-SRBs), this mutant was cultured (30 L) and the active components were purified. Two active components (KA54-SRB1 and KA54-SRB2) were detected in ESI-MS and chiral HPLC analysis. The molecular formulae for KA54-SRB1 and KA54-SRB2 are C_15_H_26_O_4_ and C_16_H_28_O_4_, whose values are one oxygen smaller and two hydrogen larger when compared with those for SRB1 and SRB2, respectively. Based on extensive NMR analysis, the signaling molecules in KA54 were determined to be 6′-deoxo-SRB1 and 6′-deoxo-SRB2. Gel shift analysis indicated that a ligand affinity of 6′-deoxo-SRB1 to the specific receptor SrrA was 100-fold less than that of SRB1. We performed bioconversion of the synthetic 6′-deoxo-SRB1 in the *Streptomyces lividans* recombinant carrying SrrO-expression plasmid. Substrate 6′-deoxo-SRB1 was converted through 6′-deoxo-6′-hydroxy-SRB1 to SRB1 in a time-dependent manner. Thus, these results clearly indicated that SrrO catalyzes the C-6′ oxidation at a final step in SRB biosynthesis.

## 1. Introduction

The soil-dwelling filamentous bacterial genus, *Streptomyces*, is well characterized by their distinct properties to produce a vast array of pharmaceutically and agriculturally important secondary metabolites. *Streptomyces* species generally produce diffusible signaling molecules that induce secondary metabolite production and morphological differentiation [1,2]. Hitherto discovered signaling molecules are classified into three groups; γ-butyrolactone-type, furan-type, and butenolide-type [3,4,5,6]. For example, γ-butyrolactone-type contains A-factor in *Streptomyces griseus* for streptomycin production [7,8], furan-type does methylenomycin furans in *Streptomyces coelicolor* for methylenomycin production [9], and butenolide-type contains avenolide in *Streptomyces avermitilis* for avermectin [10] and SRBs in *Streptomyces rochei* for lankacidin/lankamycin [11].

**Figure 1:**
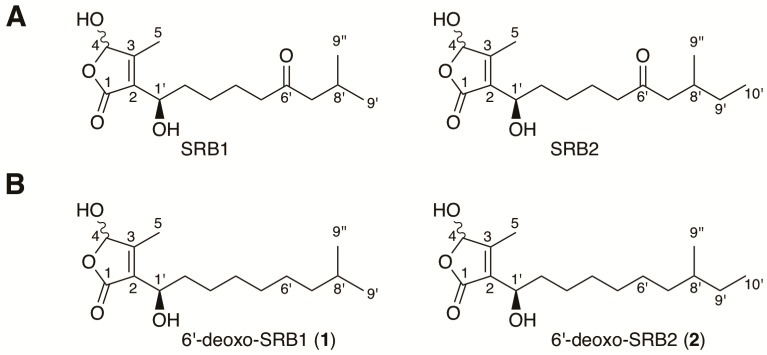
Structures of *Streptomyces rochei* signaling molecules. (**A**) natural signaling molecules SRB1 and SRB2 isolated from *S. rochei* 7434AN4; (**B**) 6′-deoxo-SRB1 (**1**) and 6′-deoxo-SRB2 (**2**) isolated from the *srrO* mutant (strain KA54) of *S. rochei.*

*Streptomyces rochei* strain 7434AN4 produces two structurally unrelated polyketide antibiotics, lankacidin and lankamycin (Figure S1), and carries three linear plasmids (pSLA2-L, -M, and -S) [12]. Lankacidin and lankamycin inhibit peptide synthesis synergistically by targeting neighboring sites in bacterial ribosome [13,14]. In addition, lankacidins could exhibit considerable antitumor activity in paclitaxel-like action [15,16]. These biosynthetic gene clusters are located on the largest giant linear plasmid pSLA2-L (210,614 bp) [17,18,19,20]. In addition, their regulatory genes including an SRB biosynthesis gene (*srrX*), an SRB receptor gene (*srrA*), and two SARP (*Streptomyces* antibiotic regulatory protein) genes (*srrY* and *srrZ*) are coded on pSLA2-L. We revealed that the signaling pathway goes from *srrX* through *srrA* to *srrY*, leading to lankacidin production [21,22]. For lankamycin production, *srrY* directly activates the transcription of *srrZ* [23]. In addition, we have elucidated the chemical structures of signaling molecules SRB1 and SRB2 in *S. rochei*; SRB1 was determined to be 2-(1’-hydroxyl-6′-oxo-8’-methylnonyl)-3-methyl-4-hydroxybut-2-en-1,4-olide (C_15_H_24_O_5_), while SRB2 was 2-(1’-hydroxyl-6′-oxo-8’-methyldecyl)-3-methyl-4-hydroxybut-2-en-1,4-olide (C_16_H_26_O_5_) (Figure 1) [11]. Their C1’ stereochemistry was determined to be *R* based on chiral HPLC analysis. It is noteworthy that several possible genes for SRB biosynthesis were found around *srrX* (*orf85*) on pSLA2-L; an NAD-dependent dehydrogenase gene *srrG* (*orf81*), a phosphatase gene *srrP* (*orf83*), a P450 monooxygenase gene *srrO* (*orf84*), and a thioesterase gene *srrH* (*orf86*).

The cytochrome P450 comprises a ferric heme-containing enzyme superfamily that catalyzes the oxygenation reaction on vast array of substrates including antibiotics, lipids, and steroids, and is widely distributed across the Kingdoms from bacteria to human [24,25,26]. One of the typical chemical reactions catalyzed by P450 enzymes is an oxidation of non-reactive C-H bonds [27]. Monooxygenase reactions are involved in the macrolide biosynthesis of erythromycin [28,29], oleandomycin [30], and lankamycin [19]. Location of *srrO* (*orf84*) gene at the neighbor of *srrX* (*orf85*) indicates that *srrO* may be involved in SRB biosynthesis in *S. rochei*. We here analyzed the function of *srrO* by gene inactivation and enzymatic bioconversion experiments, the results of which will be described in this paper.

## 2. Materials and Methods

### 2.1. Strains, Reagents, and Culture Conditions

All strains and plasmids used in this study were listed in Table S1. Strain 51252 that harbors pSLA2-L in addition to the chromosome was used as a parent strain [12]. Strain KA20, a double mutant of *srrX* and the transcriptional repressor gene *srrB*, was used as signaling-molecule indicator strain [11,21]. YM medium (0.4% yeast extract, 1.0% malt extract, and 0.4% D-glucose, pH 7.3) was used for signaling-molecule synthesis and bioassay. For protoplast preparation and SrrO-protein expression, *Streptomyces* strains were grown in YEME liquid medium [31]. Protoplasts were regenerated on R1M solid medium [32]. To construct targeting plasmids for *srrO* mutation, *E. coli* strains were grown in Luria–Bertani medium supplemented with ampicillin (100 μg/mL) when necessary. Genetic manipulations for *Streptomyces* [31] and *E. coli* [33] were performed according to the described procedures.

### 2.2. Spectroscopic Instruments

NMR spectra were recorded on ECA-500 and/or ECA-600 spectrometers (JEOL, Tokyo, Japan) equipped with a field gradient accessory. Deuteriochloroform (99.8 atom% enriched; Kanto Chemical Co., Ltd., Tokyo, Japan) was used as a solvent. Chemical shifts were recorded as a δ value based on a resident solvent signal (δ_C_ = 77.0), or an internal standard signal of tetramethylsilane (δ_H_ = 0). High resolution electrospray ionization (ESI) mass spectra were measured by a LTQ Orbitrap XL mass spectrometer (Thermo Fisher Scientific, Waltham, MA, USA). High resolution gas chromatography-time of flight mass spectra (ionization mode; CI) were acquired on a JMS-T100 GCV 4G (JEOL, Tokyo, Japan). Optical rotations were measured using a DIP-370 polarimeter (JASCO Cooperation, Tokyo, Japan). IR spectra were recorded on a IRAffiniy-1 spectrometer (Shimadzu Cooperation, Kyoto, Japan) using the ATR (Attenuated Total Reflection) method.

### 2.3. Construction of the srrO Mutant KA54

A 3.8-kb StuI-EcoRI fragment (nt 143,844,147,674 of pSLA2-L) containing an srrO gene was ligated with 2.8-kb StuI-EcoRI fragment of Litmus 28i to give pKAR3041. To a ClaI restriction site at the 5’-terminal region of srrO was introduced a 1.0-kb ClaI fragment of aac(3)IV gene cassette conferring apramycin resistance. The resulting plasmid pKAR3043 was digested with EcoRI and StuI, and the vector of which was replaced with an EcoRI-SmaI fragment of pRES18, an E. coli-Streptomyces shuttle vector [34], to afford pKAR3044. This plasmid was transformed into protoplast of strain 51252, and then an srrO mutant KA54 was obtained through homologous recombination according to our protocol [18] (Figure S2).

### 2.4. Metabolites in the srrO Mutant KA54

Antibiotic production in strain KA54 was analyzed by high performance liquid chromatography (HPLC) and thin layer chromatography (TLC) in comparison with that in parent strain 51252. The crude extract was applied on a COSMOSIL CHOLESTER column (4.6 × 250 mm, Nacalai Tesque, Kyoto, Japan) and eluted with a mixture of acetonitrile-10 mM sodium phosphate buffer (pH 8.2) (3:7, *v*/*v*) at a flow rate of 1.0 mL/min. The eluate was monitored at 230 nm with a MD-2010 multiwavelength photodiode array detector (JASCO Cooperation, Tokyo, Japan). TLC was developed with a mixture of CHCl_3_-methanol (15:1, *v*/*v*) and baked after spraying with anisaldehyde-H_2_SO_4_.

### 2.5. Isolation of Signaling Molecules from the srrO Mutant KA54

**Figure 2:**
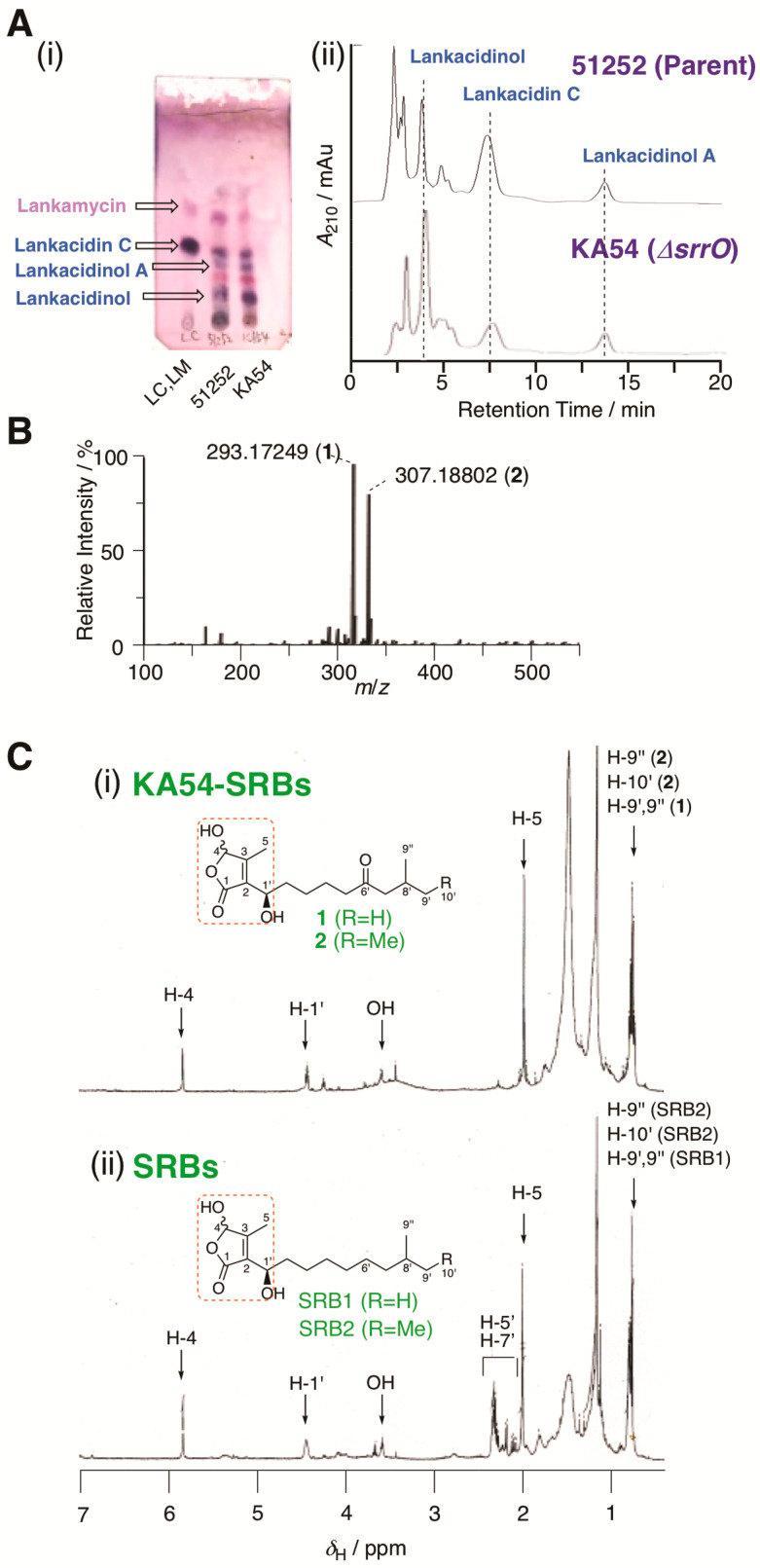
Metabolite analysis of strain KA54. (**A**) (**i**) TLC analysis of metabolites in strains KA54 (*srrO* mutant) and 51252 (parent). LC; standard sample of lankacidin C. (**ii**) HPLC analysis of metabolites in strains KA54 and 51252. Chromatogram was monitored by UV absorbance at 230 nm. Structures of lankacidin C and lankamycin were displayed in Supplementary Figure S1. (**B**) ESI-MS spectrum of KA54-SRBs (**1** and **2**). (**C**) ^1^H NMR spectrum of (**i**) KA54-SRBs (**1** and **2**) and (**ii**) SRBs.

Seed culture (20 mL) of strain KA54 was inoculated into to 2 L YM medium in 5-L Erlenmeyer flask, which was grown at 28 °C for 36 h. A total of 30 L culture broth was extracted with equal volume of EtOAc twice. The combined organic phase was dried (Na_2_SO_4_), filtered, and concentrated to dryness. The resulting crude extracts were purified by Sephadex LH-20 (GE Healthcare, Chicago, IL, USA) with methanol. Each fraction was subjected to a bioassay using strain KA20 as a test organism according to our previous report [11]. Active fractions were collected and purified by a series of silica gel column chromatography with two different solvent systems of CHCl_3_-MeOH (50:1, *v*/*v*) and toluene-EtOAc (3:1, *v*/*v*). A mixture of two active components (KA54-SRB1 and KA54-SRB2) (100 μg from 30 L culture) was analyzed by ESI-MS and NMR (Figure 2).

Compound **1** (KA54-SRB1): high resolution ESI-MS; observed *m*/*z* 293.1727 [M + Na]^+^ (calcd for C_15_H_26_O_4_Na, 293.1729).

Compound **2** (KA54-SRB2): high resolution ESI-MS; observed *m*/*z* 307.1883 [M + Na]^+^ (calcd for C_16_H_28_O_4_Na, 307.1885).

Nevertheless, the low quantity of active components (100 μg from 30 L culture), compounds **1** and **2** were separated by repeated runs of HPLC (25% aqueous acetonitrile containing 0.1% trifluoroacetic acid) at 10.6 min and 17.1 min, respectively. Their C-1’ stereochemistry was confirmed by chiral HPLC using synthetic 6′-deoxo-SRBs (details were shown in Section 2.7 and Section 3.3).

### 2.6. Synthesis of 6′-deoxo-SRBs

6-Hydroxyhexyl *p*-toluenesulfonate (**4**)

To a solution of 1,6-hexanediol (**3**) (2.03 g, 17.2 mmol), triethylamine (7.00 mL, 50.5 mmol), and 4,4-dimethylaminopyridine (50 mg, 0.41 mmol) in CH_2_Cl_2_ (70 mL) was added *p*-toluenesulfonyl chloride (3.28 g, 17.2 mmol) at 0 °C, and the mixture was stirred at room temperature for 1 h. Water (50 mL) was added, and the mixture was extracted with EtOAc twice. The combined organic phases were washed with brine, dried (Na_2_SO_4_), filtered, and concentrated to dryness. The residue was purified over silica gel with hexane-EtOAc (1:1–1:2, *v*/*v*) to give **4** (2.34 g, 50%) as a colorless oil.

^1^H-NMR (CDCl_3_): δ = 1.33 (m, 4H), 1.51 (m, 2H), 1.65 (m, 2H), 2.45 (s, 3H), 3.60 (t, *J* = 6.5 Hz, 2H), 4.02 (t, *J* = 6.3 Hz, 2H), 7.35 (d, *J* = 8.0 Hz, 2H), 7.78 ppm (d, *J* = 8.0 Hz, 2H); ^13^C-NMR (CDCl_3_): δ = 21.6, 25.0, 25.1, 28.7, 32.3, 62.5, 127.8, 129.8, 133.0, 144.7; High resolution ESI-MS: observed *m*/*z* 295.0975 [M + Na]^+^ (calcd for C_13_H_20_O_4_SNa, 295.0975); IR (neat): ν = 3343, 2934, 2860, 2361, 2342, 1354, 1172 cm^−1^.

6-((Tetrahydro-2*H*-pyran-2-yl)oxy)hexyl *p*-toluenesulfonate (**5**)

A mixture of alcohol **4** (2.30 g, 8.45 mmol), 3,4-dihydro-2*H*-pyrane (1.50 mL, 16.4 mmol), and *p*-toluenesulfonic acid monohydrate (45 mg) in CH_2_Cl_2_ (30 mL) was stirred at room temperature for 8 h. Saturated aqueous NaHCO_3_ (20 mL) was added in a dropwise manner, and the mixture was extracted with EtOAc twice. The combined organic phases were washed with brine, dried (Na_2_SO_4_), filtered, and concentrated to dryness. The residue was purified over silica gel with hexane-EtOAc (10:1, *v*/*v*) to give **5** (2.85 g, 95%) as a colorless oil.

^1^H-NMR (CDCl_3_): δ = 1.32-1.33 (m, 4H), 1.50-1.84 (m, 10H), 2.45 (s, 3H), 3.32-3.72 (m, 2H), 3.47–3.87 (m, 2H) 4.02 (t, *J* = 6.3 Hz, 2H), 4.54 (t, *J* = 3.3 Hz, 1H), 7.34 (d, *J* = 8.5 Hz, 2H), 7.78 ppm (d, *J* = 8.0 Hz, 2H); ^13^C-NMR (CDCl_3_): δ = 19.7, 21.6, 25.2, 25.4, 25.6, 28.7, 29.4, 30.7, 62.4, 67.3, 70.5, 98.9, 127.8, 129.7, 133.1, 144.6; High resolution ESI-MS: observed *m*/*z* 379.1551 [M + Na]^+^ (calcd for C_18_H_28_O_5_SNa, 379.1550); IR (neat): ν = 2938, 2862, 2361, 2342, 1354, 1175 cm^−1^.

2-((8-Methylnonyl)oxy)tetrahydro-2*H*-pyran (**6**)

To a suspension of magnesium (1.00 g, 41.1 mmol) and iodine (65 mg) in THF (20 mL) was added 1 -bromo-2-methylpropane (4.50 mL, 41.7 mmol) at 0 °C, and the mixture was stirred at 0 °C for 30 min. A solution of 0.5 M Li_2_CuCl_4_ in THF (5.00 mL, 2.50 mmol) was added to the mixture at 0 °C, and then a solution of tosylate **5** (3.10 g, 8.70 mmol) in THF (10 mL) was added dropwise to the mixture at 0 °C, and the mixture was stirred at 0 °C for 1 h. Saturated aqueous NH_4_Cl (20 mL) was added in a dropwise manner at 0 °C, and the mixture was extracted with EtOAc twice. The combined organic phases were washed with brine, dried (Na_2_SO_4_), filtered, and concentrated to dryness. The residue was purified over silica gel with hexane-EtOAc (25:1, *v*/*v*) to give **6** (2.10 g, 99%) as a colorless oil.

^1^H-NMR (CDCl_3_): δ = 0.86 (d, *J* = 7.0 Hz, 6H), 1.13-1.17 (m, 2H), 1.25–1.35 (m, 8H), 1.50–1.60 (m, 7H), 1.71 (m, 1H), 1.83 (m, 1H), 3.36–3.52 (m, 2H), 3.71–3.90 (m, 2H), 4.58 (t, *J* = 3.5 Hz, 1H); ^13^C-NMR (CDCl_3_): δ = 14.0, 19.6, 22.6, 25.5, 25.9, 29.2, 29.7, 30.7, 31.6, 33.5, 62.2, 67.6, 98.8; High resolution ESI-MS: observed *m*/*z* 265.2138 [M + Na]^+^ (calcd for C_15_H_30_O_2_Na, 265.2138); IR (neat): ν = 2924, 2853, 1466, 1366, 1352, 1130, 1117, 1034, 1022 cm^−1^.

8-Methylnonan-1-ol (**7**)

A solution of THP ether **6** (2.43 g, 10.0 mmol) and 2M aqueous HCl (5.0 mL) in THF-MeOH (40 mL, 1:1 *v*/*v*) was stirred at room temperature for 4 h. The mixture was concentrated in vacuo. The residue was purified over silica gel with hexane-EtOAc (10:1–2:1, *v*/*v*) to give alcohol **7** (1.47 g, 92%) as a colorless oil.

^1^H-NMR (CDCl_3_): δ = 0.86 (d, *J* = 7.0 Hz, 6H), 1.13–1.17 (m, 2H), 1.23–1.38 (m, 8H), 1.49–1.68 (m, 3H) 3.60 (t, *J* = 6.5 Hz, 2H); ^13^C-NMR (CDCl_3_): δ = 22.6, 25.7, 27.3, 27.9, 29.5, 29.8, 32.8, 39.0, 63.1; High resolution GC-CI-MS: observed *m*/*z* 157.1589 [M – H]^+^ (calcd for C_10_H_21_O, 157.1592); IR (neat): ν = 3329, 2955, 2928, 2859, 1458, 1057 cm^−1^.

8-Methylnonanal (**8**)

A mixture of alcohol **7** (1.02 g, 6.44 mmol), pyridinium chlorochromate (2.48 g, 11.5 mmol), and sodium acetate (228 mg, 2.78 mmol) in CH_2_Cl_2_ (50 mL) was stirred at room temperature for 2 h. The mixture was diluted with ether (70 mL), and filtered through a pad of Celite. The filtrate and washings were concentrated in vacuo. The residue was purified over silica gel with hexane-EtOAc (15:1, *v*/*v*) to give aldehyde **8** (869 mg, 86%) as a colorless oil.

^1^H-NMR (CDCl_3_): δ = 0.86 (d, *J* = 7.0 Hz, 6H), 1.16 (m, 2H), 1.26–1.33 (m, 6H), 1.51 (m, 1H), 1.63 (m, 2H), 2.42 (dt, *J* = 1.5 and 7.3 Hz, 2H), 9.76 (t, *J* = 2.0 Hz, 1H); ^13^C-NMR (CDCl_3_): δ = 22.1, 22.6, 27.1, 27.9, 29.2, 29.6, 38.9, 43.9, 203.0; High resolution GC-CI-MS: observed *m*/*z* 157.1589 [M + H]^+^ (calcd for C_10_H_21_O, 157.1592); IR (neat): ν = 2951, 2924, 2855, 1726, 1709, 1466 cm^−1^.

(1’*R*)-2-(1’-Hydroxyl-8’-methylnonyl)-3-methyl-4-(L-menthyloxy)but-2-en-1,4-olide (**10a**) and (1’*S*)-2-(1’-Hydroxyl-8’-methylnonyl)-3-methyl-4-(L-menthyloxy)but-2-en-1,4-olide (**10b**).

A solution of *n*-butyl lithium (2.10 mL, 1.64 M in hexane, 3.44 mmol) was added in a dropwise manner to a solution of diisopropylamine (470 μL, 3.34 mmol) in THF (7 mL) at −78 °C. After 30 min of stirring, hexamethylphosphoric triamide (HMPA) (2.00 mL) was added to the mixture at −78 °C. A solution of 3-methyl-4-(L-menthyloxy)but-2-en-1,4-olide (**9**) (745 mg, 2.95 mmol) [11,35,36] in THF (7 mL) was added to the mixture at −78 °C over 15 min, and the mixture was further stirred at the same temperature for 1 h. Then, a solution of aldehyde **8** (685 mg, 4.38 mmol) in THF (7 mL) was added dropwise at −78 °C over 10 min, and the mixture was further stirred at the same temperature for 1.5 h. Saturated aqueous NH_4_Cl (20 mL) was added to the mixture, and the mixture was extracted with CH_2_Cl_2_ twice. The combined organic phases were washed with brine, dried (Na_2_SO_4_), filtered, and concentrated to dryness. The residue was purified by silica gel chromatography with hexane-EtOAc (10:1–7:1, *v*/*v*) to give a 2:1 mixture of **10a** and **10b** (452 mg, 38%) as a colorless oil, which were further separated by repeated runs of flash chromatography with hexane-EtOAc (10:1, *v*/*v*). Their absolute configuration at C-1’ was established by the modified Mosher method [37].

Compound **10a**: [α]_D_^20^ = −83.1 (*c* = 0.32, CHCl_3_); ^1^H-NMR (CDCl_3_): δ = 0.86 (d, J = 6.4 Hz, 6H), 1.15 (m, 2H), 1.26–1.33 (m, 6H), 1.41–1.55 (m, 2H), 1.65 (m, 2H), 1.84 (m, 1H), 1.98 (s, 3H), 2.88 (br, 1H), 4.46 (br, *J* = 7.5 Hz, 1H), 5.70 (s, 1H), menthyl resonances: 0.81 (d, *J* = 6.8 Hz, 3H), 0.86 (m, 1H), 0.88 (d, *J* = 7.1 Hz, 3H), 0.96 (d, *J* = 6.4 Hz, 3H), 1.02 (m, 2H), 1.22–1.27 (m, 1H), 1.28–1.42 (m, 1H), 1.64–1.70 (m, 2H), 2.08–2.14 (m, 2H), 3.62 (dt, *J* = 4.3 and 11 Hz, 1H); ^13^C-NMR (CDCl_3_): δ = 11.5, 22.6, 25.5, 27.2, 27.9, 29.3, 29.7, 36.7, 38.9, 67.0, 100.8, 130.6, 155.3, 171.4, menthyl resonances: 15.9, 20.8, 22.2, 23.2, 25.3, 31.4, 34.2, 40.5, 47.7, 79.5; High resolution ESI-MS: observed *m*/*z* 409.3320 [M + H]^+^ (calcd for C_25_H_45_O_4_, 409.3312); IR (neat): ν = 2951, 2922, 2868, 2854, 1751, 1456, 1384, 1367, 1331, 1126, 1093, 945 cm^−1^.

Compound **10b**: [α]_D_^28^ = −62.5 (*c* = 0.460, CHCl_3_); ^1^H-NMR (CDCl_3_): δ = 0.86 (d, *J* = 6.7 Hz, 6H), 1.15 (m, 2H), 1.24–1.33 (m, 6H), 1.40–1.55 (m, 2H), 1.66 (m, 2H), 1.83 (m, 1H), 1.99 (s, 3H), 2.77 (br, 1H), 4.47 (br, *J* = 6.4 Hz, 1H), 5.71 (s, 1H), menthyl resonances: 0.81 (d, *J* = 6.8 Hz, 3H), 0.86 (m, 1H), 0.88 (d, *J* = 7.1 Hz, 3H), 0.96 (d, *J* = 6.4 Hz, 3H), 1.02 (m, 2H), 1.22–1.27 (m, 1H), 1.28–1.42 (m, 1H), 1.64–1.70 (m, 2H), 2.08–2.14 (m, 2H), 3.62 (dt, *J* = 4.3 and 11 Hz, 1H); ^13^C-NMR (CDCl_3_): δ = 11.5, 22.6, 25.5, 27.2, 27.9, 29.3, 29.7, 36.7, 38.9, 67.0, 100.8, 130.6, 155.3, 171.4, menthyl resonances: 15.9, 20.8, 22.2, 23.2, 25.3, 31.4, 34.2, 40.5, 47.7, 79.5; High resolution ESI-MS: observed *m*/*z* 409.3315 [M + H]^+^ (calcd for C_25_H_45_O_4_, 409.3312); IR (neat): ν = 2951, 2922, 2868, 2855, 1751, 1456, 1385, 1368, 1330, 1094, 945 cm^−1^.

6′-Deoxo-SRB1a (**1a**)

To a solution of menthyl ester **10a** (34 mg, 83.2 μmol) in CH_2_Cl_2_ (4.0 mL) was added 10% BBr_3_ solution in CH_2_Cl_2_ (400 μL, 420 μmol) at −78 °C, and the mixture was stirred at −78 °C for 3 h. Saturated aqueous NaHCO_3_ (3 mL) was carefully added, and the mixture was extracted with EtOAc twice. The combined organic phases were washed with brine, dried (Na_2_SO_4_), filtered, and concentrated to dryness. The residue was purified by silica gel chromatography with hexane-EtOAc (2:1, *v*/*v*) to give 6′-deoxo-SRB1a (**1a**) (17 mg, 79%) as a colorless oil.

Mixture of C-4 epimers: [α]_D_^18^ = +126.4 (*c* = 0.11, CHCl_3_); ^1^H-NMR (CDCl_3_): δ = 0.86 (d, *J* = 6.7 Hz, H-9’ and H-9’’, 6H), 1.15 (m, H-7’, 2H), 1.26 (m, H-3’a, H-4’, and H-6′, 5H), 1.29 (m, H-5’, 2H), 1.39 (m, H-3’b, 1H), 1.51 (m, H-8’, 1H), 1.65 (m, H-2’a, 1H), 1.81 (m, H-2’b, 1H), 2.07 (s, H-5, 3H), 4.47 (m, H-1’, 1H), 5.85 (brs, H-4, 1H); ^13^C-NMR (CDCl_3_): δ = 11.4/11.6 (C-5), 22.6 (C-9’ and C-9’’), 25.5 (C-3’), 27.3 (C-6′), 27.9 (C-8’), 29.3 (C-5’), 29.7/29.8 (C-4’), 36.0/36.2 (C-2’), 39.0 (C-7’), 66.7 (C-1’), 98.8 (C-4), 130.1/130.5 (C-2), 157.9 (C-3), 171.7/172.0 (C-1); High resolution ESI-MS: observed *m*/*z* 293.1718 [M + Na]^+^ (calcd for C_15_H_26_O_4_Na, 293.1723); IR (neat): *ν* = 3399, 2957, 2924, 2855, 2361, 2342, 1749, 1734 cm^−1^.

6′-Deoxo-SRB1b (**1b**)

The compound **10b** (20 mg, 49 μmol) was treated in the same manner as described for the preparation of **1a** to give 6′-deoxo-SRB1b (**1b**) (8.1 mg, 62%) as a colorless oil.

Mixture of C-4 epimers: [α]_D_^25^ = +1.32 (*c* = 0.180, CHCl_3_); ^1^H-NMR (CDCl_3_): δ = 0.86 (d, *J* = 6.7 Hz, H-9’ and H-9’’, 6H), 1.15 (m, H-7’, 2H), 1.26 (m, H-3’a, H-4’, and H-6′, 5H), 1.29 (m, H-5’, 2H), 1.39 (m, H-3’b, 1H), 1.51 (m, H-8’, 1H), 1.65 (m, H-2’a, 1H), 1.81 (m, H-2’b, 1H), 2.07 (s, H-5, 3H), 4.47 (m, H-1’, 1H), 5.85 (brs, H-4, 1H); ^13^C-NMR (CDCl_3_): δ = 11.4/11.6 (C-5), 22.6 (C-9’ and C-9’’), 25.5 (C-3’), 27.3 (C-6′), 27.9 (C-8’), 29.3 (C-5’), 29.8 (C-4’), 36.0/36.4 (C-2’), 39.0 (C-7’), 66.8 (C-1’), 98.7/98.8 (C-4), 130.1/130.6 (C-2), 157.4 (C-3), 171.6/171.7 (C-1); High resolution ESI-MS: observed *m*/*z* 293.1720 [M + Na]^+^ (calcd for C_15_H_26_O_4_Na, 293.1723); IR (neat): *ν* = 3361, 2951, 2924, 2855, 1743, 1466 cm^−1^.

2-(((S)-8-Methyldecyl)oxy)tetrahydro-2*H*-pyran (**11**)

A suspension of (*S*)-(+)-1-chloro-2-methylbutane (5.00 mL, 41.7 mmol), magnesium (1.01 g, 41.7 mmol), and iodine (50 mg) in THF (20 mL) was refluxed for 45 min. A solution of 0.5 M Li_2_CuCl_4_ solution in THF (5.00 mL, 2.50 mmol) was added to the mixture at 0 °C, and then a solution of tosylate **5** (2.31 g, 6.48 mmol) in THF (9 mL) was added dropwise to the mixture at 0 °C, and the mixture was stirred at 0 °C for 1 h. Saturated aqueous NH_4_Cl (20 mL) was added in a dropwise manner at 0 °C, and the mixture was extracted with EtOAc twice. The combined organic phases were washed with brine, dried (Na_2_SO_4_), filtered, and concentrated to dryness. The residue was purified over silica gel with hexane-EtOAc (25:1, *v*/*v*) to give THP-ether **11** (1.65 g, 99%) as a colorless oil.

Diastereomer mixture: ^1^H-NMR (CDCl_3_): δ = 0.83 (d, *J* = 6.4 Hz, 3H), 0.85 (t, *J* = 6.7 Hz, 3H), 1.12 (m, 2H), 1.24–1.36 (m, 12H), 1.53–1.61 (m, 5H), 1.72 (m, 1H), 1.83 (m, 1H), 3.36–3.52 (m, 2H), 3.71–3.90 (m, 2H), 4.58 (t, *J* = 3.5 Hz, 1H), 5.70 (s, 1H); ^13^C-NMR (CDCl_3_): δ = 11.4, 19.2, 19.7, 25.5, 26.2, 27.0, 29.5, 29.5, 29.8, 29.9, 30.8, 34.4, 36.6, 62.3, 67.7, 98.8; High resolution ESI-MS: observed *m*/*z* 279.2295 [M + Na]^+^ (calcd for C_16_H_32_O_2_Na, 279.2295); IR (neat): *ν* = 2955, 2924, 2852, 1458, 1377, 1034, 1022 cm^−1^.

(*S*)-8-Methyldecan-1-ol (**12**)

The THP-ether **11** (1.65 g, 6.43 mmol) was treated in the same manner as described for the preparation of **7** to give alcohol **12** (1.10 g, 99%) as a colorless oil.

[α]_D_^26^ = +3.50 (*c* = 1.00, CHCl_3_); ^1^H-NMR (CDCl_3_): δ = 0.84 (d, *J* = 6.1 Hz, 3H), 0.85 (t, *J* = 7.3 Hz, 3H), 1.07–1.16 (m, 2H), 1.26-1.36 (m, 11H), 1.56 (m, 2H), 3.62 (t, J=6.7, 2H); ^13^C-NMR (CDCl_3_): δ = 11.3, 19.1, 25.7, 27.0, 29.4, 29.9, 32.7, 34.3, 36.6, 62.9; High resolution GC-CI-MS: observed *m*/*z* 171.1744 [M + Na]^+^ (calcd for C_11_H_23_O, 171.1749); IR (neat): *ν* = 3318, 2959, 2924, 2853, 1458, 1377, 1055 cm^−1^.

(*S*)-8-Methyldecanal (**13**)

The compound **12** (810 mg, 4.70 mmol) was treated in the same manner as described for the preparation of **8** to give **13** (746 mg, 93%) as a colorless oil.

[α]_D_^27^ = +0.75 (*c* = 1.00, CHCl_3_); ^1^H-NMR (CDCl_3_): δ = 0.84 (d, *J* = 6.5 Hz, 3H), 0.85 (t, *J* = 7.0 Hz, 3H), 1.07–1.16 (m, 2H), 1.24–1.30 (m, 9H), 1.62 (m, 2H), 2.42 (dt, *J* = 2.4 and 7.3, 2H), 9.77 (t, *J* = 2.4 Hz, 1H); ^13^C-NMR (CDCl_3_): δ = 11.4, 19.2, 22.1, 26.9, 29.2, 29.5, 29.7, 34.4, 36.5, 43.9, 203.0; High resolution GC-CI-MS: observed *m*/*z* 171.1751 [M + H]^+^ (calcd for C_10_H_23_O, 171.1749); IR (neat): *ν* = 2957, 2924, 2855, 1707, 1458, 1412, 1287 cm^−1^.

(1’*R*,8*S*)-2-(1’-Hydroxyl-8’-methyldecyl)-3-methyl-4-(L-menthyloxy)but-2-en-1,4-olide (**14a**) and (1’*S*,8*S*)-2-(1’-Hydroxyl-8’-methyldecyl)-3-methyl-4-(L-menthyloxy)but-2-en-1,4-olide (**14b**).

Compound **13** (696 mg, 4.09 mmol) and L-menthyloxy-butenolide (962 mg, 3.81 mmol) were treated in the same manner as described for the preparation of **10** to give a 2:1 mixture of **14a** and **14b** (806 mg, 50%) as a colorless oil, which was also further separated by flash chromatography.

Compound **14a**: [α]_D_^23^ = −70.7 (*c* = 1.71, CHCl_3_); ^1^H-NMR (CDCl_3_): δ = 0.84 (d, *J* = 6.4 Hz, 3H), 0.85 (d, *J* = 7.0 Hz, 3H), 1.24–1.34 (m, 10H), 1.63–1.66 (m, 2H), 1.84 (m, 1H), 1.98 (s, 3H), 2.83 (br, 1H), 4.45 (br, *J* = 7.5 Hz, 1H), 5.70 (s, 1H), menthyl resonances: 0.81 (d, *J* = 6.8 Hz, 3H), 0.86 (m, 1H), 0.88 (d, *J* = 7.1 Hz, 3H), 0.96 (d, *J* = 6.4 Hz, 3H), 1.02 (m, 2H), 1.22–1.27 (m, 1H), 1.28–1.42 (m, 1H), 1.64–1.70 (m, 2H), 2.08–2.14 (m, 2H), 3.62 (dt, *J* = 4.3 and 11 Hz, 1H); ^13^C-NMR (CDCl_3_): δ = 11.4, 11.5, 19.2, 25.5, 27.0, 29.4, 29.5, 29.9, 34.3, 36.6, 36.7, 67.0, 100.9, 130.6, 155.3, 171.4, menthyl resonances: 15.9, 20.8, 22.2, 23.2, 25.4, 31.5, 34.2, 40.5, 47.7, 79.5; High resolution ESI-MS: observed *m*/*z* 445.3289 [M + Na]^+^ (calcd for C_26_H_46_O_4_Na, 445.3288); IR (neat): *ν* = 2953, 2922, 2868, 2854, 1751, 1456, 1331, 1096, 943 cm^−1^.

Compound **14b**: [α]_D_^22^ = −95.4 (*c* = 1.00, CHCl_3_). ^1^H-NMR (CDCl_3_): ^1^H-NMR (CDCl_3_): δ = 0.84 (d, *J* = 6.4, 3H), 0.85 (d, *J* = 7.0, 3H), 1.24-1.34 (m, 10H), 1.63 (m, 2H), 1.83 (m, 1H), 1.98 (s, 3H), 2.77 (br, 1H), 4.47 (br, 1H), 5.71 (s, 1H), menthyl resonances: 0.81 (d, *J* = 6.8 Hz, 3H), 0.86 (m, 1H), 0.88 (d, *J* = 7.1 Hz, 3H), 0.96 (d, *J* = 6.4 Hz, 3H), 1.02 (m, 2H), 1.22-1.27 (m, 1H), 1.28-1.42 (m, 1H), 1.64-1.70 (m, 2H), 2.08-2.14 (m, 2H), 3.62 (dt, *J* = 4.3 and 11 Hz, 1H); ^13^C-NMR (CDCl_3_): δ = 11.4, 11.5, 19.2, 25.6, 27.0, 29.4, 29.5, 29.9, 34.3, 36.5, 66.9, 100.7, 130.7, 155.3, 171.4, menthyl resonances: 15.7, 20.9, 22.2, 23.1, 25.2, 31.4, 34.2, 40.4, 47.7, 79.5; High resolution ESI-MS: observed *m*/*z* 445.3287 [M + Na]^+^ (calcd for C_26_H_46_O_4_Na, 445.3288); IR (neat): *ν* = 2953, 2922, 2868, 2855, 1751, 1456, 1369, 1331, 1096, 943 cm^−1^.

6′-Deoxo-SRB2a (**2a**)

The compound **14a** (19 mg, 45 μmol) was treated in the same manner as described for the preparation of **1a** to give 6′-deoxo-SRB2a (**2a**) (10 mg, 80%) as a colorless oil.

Mixture of C-4 epimers: [α]_D_^20^ = +78.3 (*c* = 0.180, CHCl_3_); ^1^H-NMR (CDCl_3_): δ = 0.83 (d, *J* = 6.4 Hz, H-9’’, 3H), 0.86 (t, *J* = 6.7 Hz, H-10’, 3H), 1.07 (m, H-7’a, 1H), 1.12 (m, H-9’a, 1H), 1.28 (m, H-3’a, H-4’, H-5’, H-6′, H-7’b, H-8’, and H-9’b, 10H), 1.40 (m, H-3’b, 1H), 1.66 (m, H-2’a, 1H), 1.81 (br, H-2’b, 1H), 2.07 (s, H-5, 3H), 4.46 (m, H-1’, 1H), 5.85 (brs, H-4, 1H); ^13^C-NMR (CDCl_3_): δ = 11.4 (C-10’), 11.4/11.5 (C-5), 19.2 (C-9’’), 25.5 (C-3’), 27.0 (C-6′), 29.4 (C-5’), 29.5 (C-9’), 29.9 (C-4’), 34.4 (C-8’), 36.2 (C-2’), 36.6 (C-7’), 66.8 (C-1’), 98.6/98.7 (C-4), 130.2 (C-2), 157.2 (C-3), 171.5 (C-1); High resolution ESI-MS: observed *m*/*z* 307.1881 [M + Na]^+^ (calcd for C_16_H_28_O_4_Na, 307.1880); IR (neat): *ν* = 3385, 2955, 2924, 2855, 1736 cm^−1^.

6′-Deoxo-SRB2b (**2b**)

The compound **14b** (15 mg, 36 μmol) was treated in the same manner as described for the preparation of **1a** to give 6′-deoxo-SRB2b (**2b**) (7.3 mg, 72%) as a colorless oil.

Mixture of C-4 epimers: [α]_D_^20^ = –5.09 (*c* = 0.530, CHCl_3_); ^1^H-NMR (CDCl_3_): δ = 0.83 (d, *J* = 6.4 Hz, H-9’’, 3H), 0.86 (t, *J* = 6.7 Hz, H-10’, 3H), 1.07 (m, H-7’a, 1H), 1.12 (m, H-9’a, 1H), 1.28 (m, H-3’a, H-4’, H-5’, H-6′, H-7’b, H-8’, and H-9’b, 10H), 1.40 (m, H-3’b, 1H), 1.66 (m, H-2’a, 1H), 1.81 (br, H-2’b, 1H), 2.07 (s, H-5, 3H), 4.46 (m, H-1’, 1H), 5.85 (brs, H-4, 1H); ^13^C-NMR (CDCl_3_): δ = 11.4 (C-10’), 11.4/11.5 (C-5), 19.2 (C-9’’), 25.5 (C-3’), 27.0 (C-6′), 29.3 (C-5’), 29.5 (C-9’), 29.8 (C-4’), 34.3 (C-8’), 36.1/36.4 (C-2’), 36.6 (C-7’), 66.8 (C-1’), 98.5/98.7 (C-4), 130.2/130.7 (C-2), 157.1 (C-3), 171.5 (C-1); High resolution ESI-MS: observed *m*/*z* 307.1880 [M + Na]^+^ (calcd for C_16_H_28_O_4_Na, 307.1880); IR (neat): *ν* = 3385, 2955, 2924, 2854, 1736, 1458 cm^−1^.

### 2.7. Chiral HPLC Analysis

Natural KA54-SRB1 and KA54-SRB2 were analyzed by chiral HPLC using a Chiral MB-S column (4.6 I.D. × 250 mm, macroporous silica gel coated with optically active *N*-substituted polymaleimides; Tokyo Kasei, Co. Ltd., Tokyo, Japan). The mobile phase was composed of two solvents; 20% aqueous acetonitrile with 0.1% trifluoroacetic acid (solvent A) and 10% aqueous acetonitrile with 0.1% trifluoroacetic acid (solvent B), and samples were eluted at a flow rate of 1.0 mL/min with detection at 210 nm. The linear-gradient elution program was set as follows: 100% A (0–10 min), 0–100% A (10–25 min), 0–100% A (25–35 min) and 100% A (35–60 min). The injection volume of each sample was 10 μL. Natural 6′-deoxo-SRB1 (**1**) and 6′-deoxo-SRB2 (**2**) were eluted at 21.1 and 53.3 min, respectively. Synthetic **1a**, **1b**, **2a**, and **2b** were eluted at 21.1, 20.2, 53.3, and 50.0 min, respectively.

### 2.8. Gel Shift Assay

Preparation of an SrrA protein (SRB receptor) and a DNA probe containing the promoter region of *srrY* (*srrYp*), a target gene of SrrA, was described previously [22,38]. The reaction mixture contained the binding buffer (20 mM Tris-HCl [pH 8.0], 100 mM NaCl, 1 mM dithiothreitol, 0.1 mg of bovine serum albumin and 5% glycerol), 0.35 nM labeled DNA, and 2 μM SrrA protein. To analyze the ligand affinity of 6′-deoxo-SRB on the binding of SrrA, various concentrations of synthetic 6′-deoxo-SRBs and SRBs were added to the reaction mixture.

### 2.9. Preparation of the Streptomyces Lividans Recombinant for SrrO Protein

A 1.2-kb PCR fragment containing *srrO* (nt 145,325-144,081 complement of pSLA2-L) was amplified using the template cosmid C7 [17] and two primers, NT-srrO-OE-F and NT-srrO-OE-R (Table S1). The PCR fragment was digested with *Nde*I and *Hin*DIII and cloned into pKAR3063H [23], a (His)_6_-tag containing derivative of a constitutive expression vector pHSA81 (Michihiko Kobayashi and Yoshiteru Hashimoto, personal communication), to give pNTT01 (Figure S3). The *Streptomyces lividans* TK64 recombinant harboring pNTT01 was grown at 28 °C for 72 h in YEME liquid medium (34% sucrose) containing 10 μg/mL of thiostrepton.

### 2.10. Bioconversion of 6′-deoxo-SRB1 in the SrrO Recombinant

To a 2-day-growth culture (100 mL) of *S. lividans* TK64/pNTT01 (+SrrO) was added 2 μmol of the substrate, and the fed cultures were further incubated for 0–5 h periods. The culture supernatant was extracted with EtOAc twice, and the combined organic phase was dried (Na_2_SO_4_), filtered, and concentrated to dryness. The crude extracts were analyzed by ESI-MS and TLC. The cell culture of *S. lividans* TK64/pHSA81 (control) [39] was used for negative control.

## 3. Results

### 3.1. Construction and Metabolite Analysis of an srrO Mutant KA54

The P450 monooxygenase gene *srrO* was inactivated to analyze its function in SRB biosynthesis. An apramycin resistance gene cassette was introduced into a 5’-terminal region of *srrO*, and double crossover mutants were obtained through homologous recombination. Gene replacement was confirmed by Southern hybridization experiment. As shown in Figure S1, a 4.4-kb *Bsp*EI-*Xho*I fragment in parent 51252 was changed into two fragments with 2.3-kb and 3.1-kb in mutant KA54. A metabolite profile in strain KA54 was analyzed in comparison with that in parent 51252. KA54 produced lankacidin and lankamycin in a comparative level to 51252 (Figure 2A). There are two possibilities to explain that the disruption of the *srrO* showed no effect on the production of lankacidin and lankamycin; (1) SrrO is not involved in SRB biosynthesis, and (2) the signaling molecule(s) in the *srrO* mutant has(have) an ability to induce lankacidin and lankamycin production.

### 3.2. Structural Elucidation of Signaling Molecules in KA54

To investigate these possibilities mentioned above, we carried out the isolation of signaling molecules in KA54 (termed as KA54-SRBs). Cultivation of KA54 was stopped at a 36 h period to reduce the accumulation of lankacidin and lankamycin, which would disturb the SRB bioassay. A 30-L culture was extracted with ethyl acetate (EtOAc), and the resulting oil was purified by Sephadex LH20 with methanol. Each fraction was subjected to bioassay to check its antibiotic-inducing activity. The *srrX* mutant was used for an indicator strain that could restore lankacidin and lankamycin production in the presence of SRBs. The active fractions were combined and purified by series of silica gel chromatography with CHCl_3_-methanol (50:1, *v*/*v*) and toluene-EtOAc (3:1, *v*/*v*).

The active fractions were further analyzed by ESI-MS analysis. ESI-MS indicated the presence of two active components (KA54-SRB1 and KA54-SRB2) in the ratio 1:1 (Figure 2B). The molecular formulae for KA54-SRB1 and KA54-SRB2 were established to be C_15_H_26_O_4_ and C_16_H_28_O_4_, whose values were one oxygen smaller and two hydrogen larger when compared with those for SRB1 and SRB2, respectively. Due to the low amounts of active components KA54-SRB1 and KA54-SRB2, we further analyzed their structural assignments as a mixture (compound with C_15_H_26_O_4_ was termed as KA54-SRB1, while C_16_H_28_O_4_ as KA54-SRB2).

The NMR spectra of KA54-SRBs were measured and compared with those of SRB1 and SRB2 in parent 51252. Based on ESI-MS analysis, one oxygen atom was replaced with two hydrogens in KA54-SRBs. In ^1^H NMR of KA54-SRBs, three signals including a highly deshielded singlet methine proton at δ_H_ 5.87, a deshielded methine proton at δ_H_ 4.48, and a singlet methyl proton at δ_H_ 2.07 were conserved when compared with those in natural SRBs (Figure 2C), suggesting the presence of a 2-(1’-hydroxyl-alkyl)-3-methyl-4-hydroxybut-2-en-1,4-olide skeleton (red-color dashed blanket in Figure 2C). This butenolide skeleton was further confirmed by a 2D NMR technique including HMQC and HMBC spectra, which showed a good agreement with our previous report for natural SRBs [11]. Regarding the alkyl side chain branched at C-2, methylene proton signals at δ_H_ 2.26 (doublet), 2.18 (double-doublet), and 2.37–2.40 (multiplet) for C-5’ and C-7’ in natural SRBs could not be detected in KA54-SRBs (Figure 2C). Furthermore, carbonyl carbon at δ_C_ 213.1 in natural SRBs could not be detected in HMBC and ^13^C NMR spectra of KA54-SRBs, suggesting the replacement of C-6′ ketone with methylene in KA54-SRBs. Thus, KA54-SRB1 and KA54-SRB2 were assigned to be 2-(1’-hydroxyl-8’-methylnonyl)-3-methyl-4-hydroxybut-2-en-1,4-olide (6′-deoxo-SRB1; compound **1**) and 2-(1’-hydroxyl-8’-methyldecyl)-3-methyl-4-hydroxybut-2-en-1,4-olide (6′-deoxo-SRB2; compound **2**) (Figure 1).

### 3.3. Synthesis of 6′-deoxo-SRBs

**Scheme 1:**
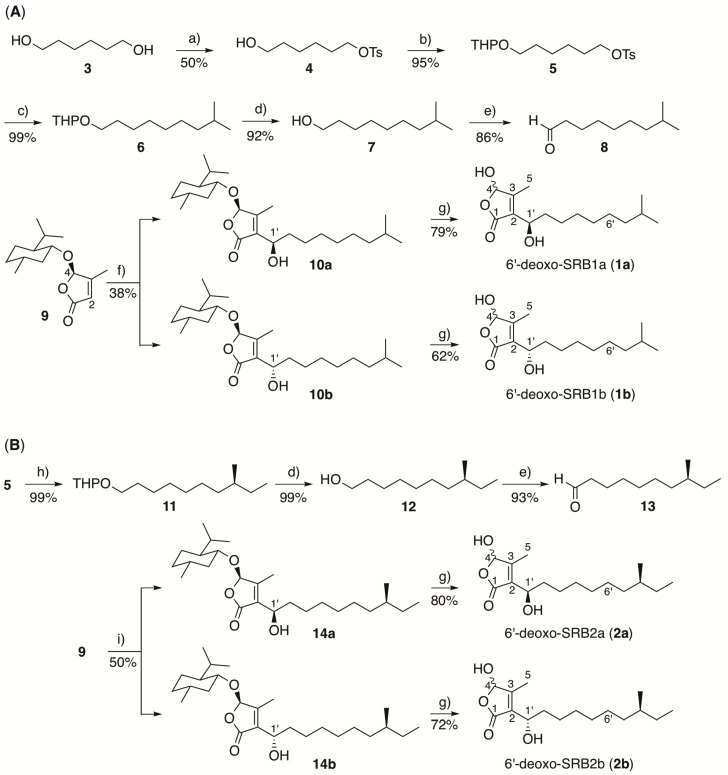
Synthesis of SRB intermediates, (**A**) 6′-deoxo-SRB1 and (**B**) 6′-deoxo-SRB2. a) *p*-toluenesulfonyl chloride, Et_3_N, 4,4-dimethylaminopyridine, CH_2_Cl_2_; b) 3,4-dihydro-2*H*-pyran, *p*-toluenesulfonic acid, CH_2_Cl_2_; c) isobutylmagnesium bromide, Li_2_CuCl_4_, THF; d) 2M HCl, THF-MeOH (1:1, *v*/*v*); e) pyridinium chlorochromate, CH_2_Cl_2_; f) LDA, THF-HMPA, and then **8**; g) BBr_3_, CH_2_Cl_2_; h) (*S*)-(2-methylbutyl)magnesium chloride, Li_2_CuCl_4_, THF; i) LDA, THF-HMPA, and then **13**.

In order to confirm the proposed structures of **1** and **2**, the (1’*R*)-isomers (**1a** and **2a**) and the (1’*S*)-isomers (**1b** and **2b**) were synthesized (Scheme 1). The commercially available 1,6-hexanediol (**3**) was treated with 1 equivalent of *p*-toluenesulfonyl chloride to give monotosylate **4**, which was then converted to tetrahydropyranyl ether **5** in 95% yield. Cross coupling of compound **5** with Grignard reagent isobutylmagnesium bromide in the presence of Li_2_CuCl_4_ [40,41] generated a C_10_ unit, which was hydrolyzed with aqueous HCl to form isodecanol **7** in 91% yield (2 steps). Alcohol **7** was subsequently oxidized with pyridinium chlorochromate (PCC) to give aldehyde **8** in 86% yield, which was then coupled with enantiomerically pure 3-methyl-4-(L-menthyloxy)but-2-en-1,4-olide (**9**) [11,35,36] in the presence of lithium diisopropylamide (LDA) to give a diastereomeric mixture of **10a** and **10b** in the ratio 2:1 in 38% yield. They were separated by repeated runs of flash silica gel chromatography to obtain compounds **10a** and **10b** with over 95% diastereomeric excess based on the peak intensities of hemiacetal H-4 proton signals (δ_H_ 5.69 for **10a** and δ_H_ 5.71 for **10b**). In addition, the C-1’ configuration was determined by the modified Mosher ester method [37]. These isomers **10a** and **10b** were separately treated with boron tribromide to form (1’*R*)-6′-deoxo-SRB1 (**1a**) and (1’*S*)-6′-deoxo-SRB1 (**1b**) in 79% and 62% yields, respectively.

6′-deoxo-SRB2a (**2a**) and 6′-deoxo-SRB2b (**2b**) were synthesized in a similar manipulation for **1a** and **1b**, except for a Grignard reagent, (*S*)-(2-methylbutyl)magnesium chloride, to form a C_11_ unit **11** (Scheme 1B).

**Figure 3:**
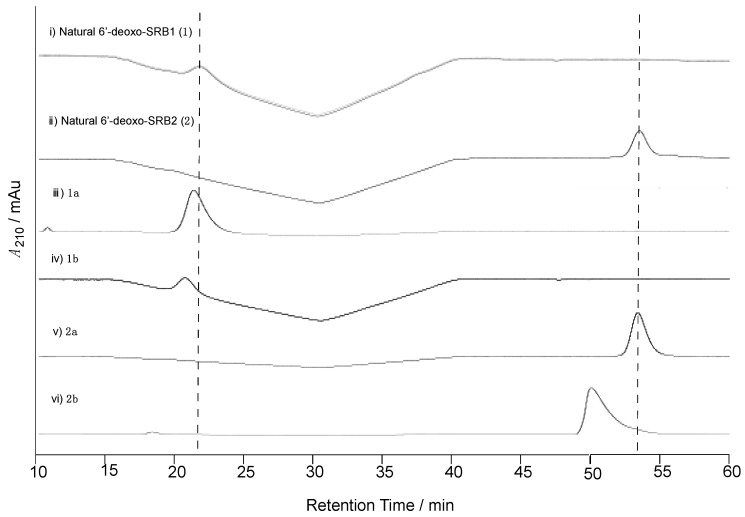
Determination of the C-1’ configuration of natural 6′-deoxo-SRBs (**1** and **2**) isolated from strain KA54. Chiral HPLC analysis of (**i**) natural 6′-deoxo-SRB1 (**1**), (**ii**) natural 6′-deoxo-SRB2 (**2**), (**iii**) synthetic **1a**, (**iv**) **1b**, (**v**) **2a**, and (**vi**) **2b**. Elution profiles were monitored by UV absorbance at 210 nm.

To confirm the C-1’ stereochemistry in compounds **1** and **2**, chiral HPLC analysis was performed (Figure 3). The retention times of **1** and **2** (21.1 and 53.3 min, respectively) on a chiral HPLC column were identical to those of the synthetic (1’*R*)-isomers **1a** and **2a**, whereas the synthetic (1’*S*)-isomers **1b** and **2b** eluted slightly earlier at 20.2 and 50.0 min. Thus, the C-1’ stereochemistry in 6′-deoxo-SRBs was the same as with SRBs.

### 3.4. Ligand Affinity of 6′-deoxo-SRBs

**Figure 4:**
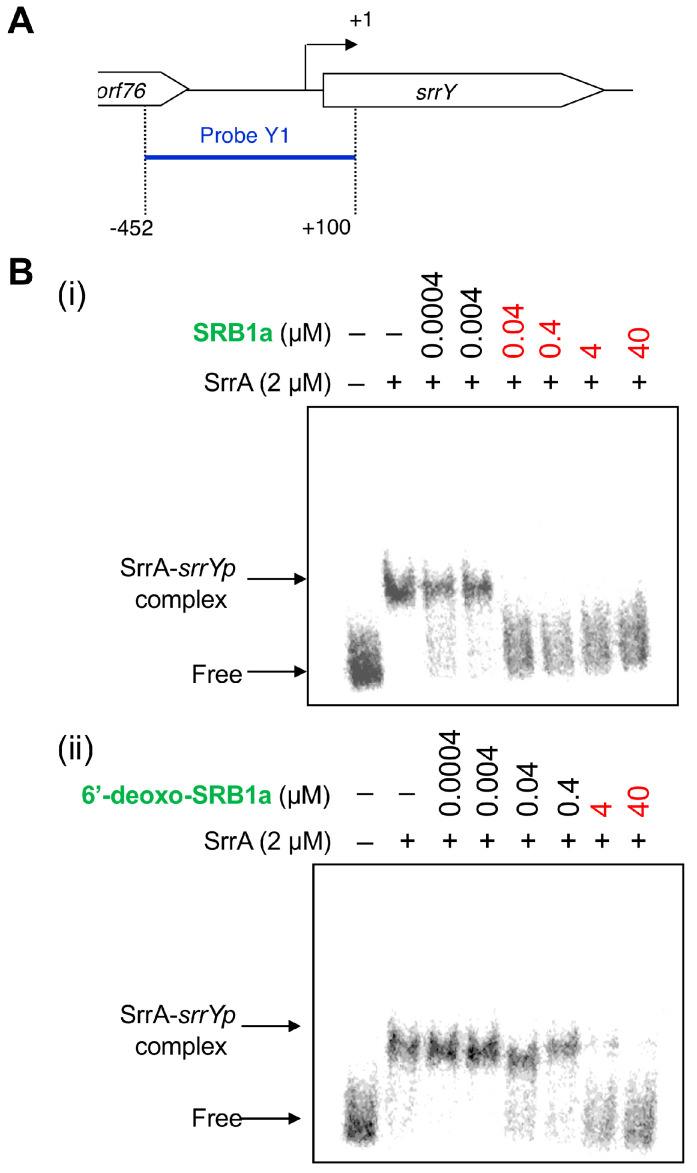
Gel shift assay of SrrA-binding in the upstream region of *srrY*. (**A**) Location of probe Y1 containing the *srrY*-promoter region (*srrYp*). The description of probe preparation was described previously [22]. (**B**) Effect of the SRB1 (**i**) and 6′-deoxo-SRB1 (**ii**) on the binding of SrrA. Each reaction mixture contained 0.35 nM probe Y1 and 2 μM recombinant SrrA protein. To the reaction mixture, various concentrations of either synthetic SRB1 ((1’*R*)-isomer; Figure 1B) [11] or synthetic 6′-deoxo-SRB1 ((1’*R*)-isomer; Figure 1B) was added.

To compare an antibiotic-inducing activity of SRBs and 6′-deoxo-SRBs, a bioassay method using the srrX mutant was not suitable since 6′-deoxo-SRBs could be converted to SRBs in some extent by SrrO protein expressed in the srrX mutant. Therefore, the antibiotic-inducing activity of 6′-deoxo-SRBs was evaluated by minimum concentration to dissociate SrrA protein from a main target gene srrY, which encodes SARP and activates lankacidin and lankamycin production in S. rochei [22]. Gel shift assay was performed using a recombinant SrrA protein and a ^32^P-labeled DNA probe containing srrY-promoter (srrYp) region in the presence of either 6′-deoxo-SRB1 or SRB1 (Figure 4A). As shown in Figure 4B, minimum concentration of 6′-deoxo-SRB1 was 100-fold higher than that of SRB1. This finding indicated that C-6′ keto group is important for improvement of antibiotic-inducing activity in S. rochei.

### 3.5. Enzymatic Bioconversion of 6′-deoxo-SRBs by SrrO Protein

**Figure 5:**
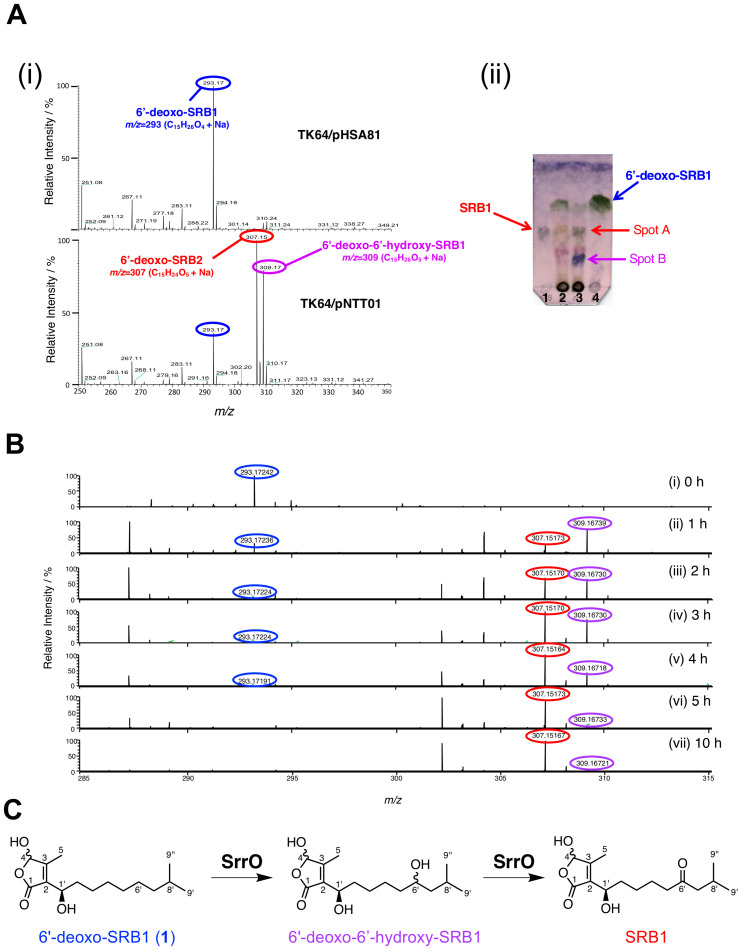
Enzymatic conversion of 6′-deoxo-SRB1 (**1**) by P450 monooxygenase SrrO in the *Streptomyces lividans* TK64/pNTT01 recombinant. (**A**) (**i**) ESI-MS spectra of reaction mixture in *S. lividans* TK64/pNTT01 (+SrrO) (upper panel) and *S. lividans* TK64/pHSA81 (control). (**ii**) TLC analysis of reaction mixture. Lane 1, the synthetic SRB1, lane 2, reaction mixture in *S. lividans* TK64/pHSA81; lane 3, reaction mixture in *S. lividans* TK64/pNTT01; lane 4, the synthetic 6′-deoxo-SRB1. TLC was developed with hexane-EtOAc = 1:2 (*v*/*v*), and baked after staining with anisaldehyde. (**B**) ESI-MS spectra of time-course enzymatic conversion of 6′-deoxo-SRB1. Samples were collected at 0, 1, 2, 3, 4, 5, and 10 h periods. (**C**) Scheme for two-stage oxidation of 6′-deoxo-SRB1 by SrrO.

To examine the enzymatic conversion of 6′-deoxo-SRBs, we constructed SrrO expression system in *Streptomyces*. The *srrO* gene was cloned into pKAR3063H [23], a (His)_6_-tag derivative of streptomycete constitutive expression vector pHSA81 (Michihiko Kobayashi and Yoshiteru Hashimoto, personal communication), to afford pNTT01 (Figure S3A). This plasmid was transformed into the heterologous host, *Streptomyces lividans* TK64, to obtain the recombinant strain. Protein expression of (His)_6_-SrrO with 45-kDa in size was confirmed by SDS-PAGE (Figure S3B). Most *Streptomyces* P450 enzymes associate with electron-recycling redox partners, ferredoxin/ferredoxin reductase, and they flexibly accept heterologous redox partners in other *Streptomyces* [42]. We therefore performed enzymatic bioconversion of 6′-deoxo-SRB1 (**1**) by the recombinant SrrO protein with the help of heterologous *S. lividans* redox partners. In an ESI-MS analysis (Figure 5A), two molecular ion peaks at *m*/*z* 307 and 309 were detected in the recombinant *S. lividans* TK64/pNTT01. In a TLC analysis (Figure 5B), compound **1** (Rf = 0.8 in hexane-EtOAc = 1:2) was converted to two compounds in the recombinant *S. lividans* TK64/pNTT01 (lane 2); spot A showed a same Rf value with synthetic SRB1 (Rf = 0.5 in hexane-EtOAc = 1:2), while another spot B did lower Rf value (Rf = 0.2 in hexane-EtOAc = 1:2). Time-dependent oxidation of compound **1** was observed in ESI-MS spectra (Figure 5C). Molecular ion peak at 293 ([M + Na]^+^ = C_15_H_26_O_4_Na) for **1** diminished in a time-dependent manner (Panels i-vii), while that at 309 ([M + Na]^+^ = C_15_H_26_O_5_Na) appeared as a major peak at the 1 h period (Panel ii). After 2 h periods, molecular ion peak at 307 ([M + Na]^+^ = C_15_H_24_O_5_Na) corresponding to SRB1 became a major peak (Panel iii). Based on the C-6′ oxidation degree in SRB biosynthesis, an intermediate with molecular ion peak at 309 was estimated to be 6′-deoxo-6′-hydroxy-SRB1 (Figure 5C), which possibly corresponded to spot B with lower Rf value on TLC (Figure 5B). Thus, SrrO converts 6′-deoxo-SRB1 into SRB1 via 6′-deoxo-6′-hydroxy-SRB1.

**Figure 6:**
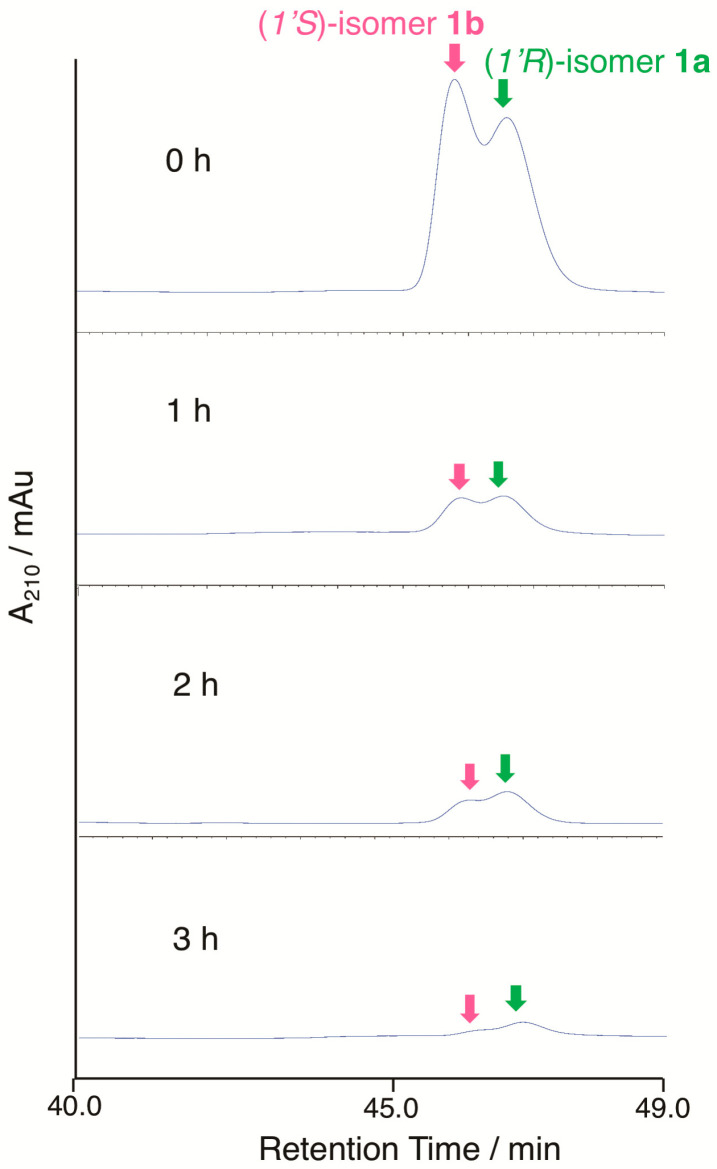
Substrate recognition of the C-1’ stereochemistry by SrrO. Conversion efficiency of a mixture of (1’*R*)-isomer **1a** and (1’*S*)-isomer **1b** was analyzed by chiral HPLC. Chromatogram was monitored by UV absorbance at 210 nm.

To judge the substrate preference on the C-1’ configuration, enzymatic conversion was performed on a mixture of (1’*R*)-6′-deoxo-SRB1 (**1a**) and (1’*S*)-6′-deoxo-SRB1 (**1b**) (Figure 6). In chiral HPLC analysis, a slightly rapid decrease of peak intensity (around 1.3-fold) at 44.4 min for **1b** was detected when compared with that at 45.1 min for **1a**. This result indicated that SrrO slightly prefers unnatural (1’*S*)-isomer **1b** to a natural (1’*R*)-isomer **1a**.

## 4. Discussion

The function of the P450 monooxygenase gene *srrO* in SRB biosynthesis was analyzed through gene disruption, gel-shift assay, and by in vivo enzymatic conversion. The *srrO* disruptant produced lankacidin and lankamycin in a comparative yield with the parent 51252, and accumulated novel signaling molecules, 6′-deoxo-SRB1 (**1** = **1a**) and 6′-deoxo-SRB2 (**2** = **2a**). Based on a ligand activity of signaling molecules for dissociation of their receptor SrrA, 6′-deoxo-SRB1 exhibits the 100-fold less binding activity compared with SRB1. Nevertheless, 6′-deoxo-SRBs could also bind to the specific SRB receptor SrrA to induce lankacidin and lankamycin production in *S. rochei*. At this stage, we have no answer why oxidation of C-6′ methylene to ketone by SrrO took place at the final step in SRB biosynthesis. One of the plausible reasons is an increase of hydrophilic property in SRB to improve antibiotic-inducing activity. To our knowledge, this is a first report to obtain biosynthetic intermediates of signaling molecules in *Streptomyces* species.

SrrO protein catalyzes two-stage oxidation of 6′-deoxo-SRBs through 6′-deoxo-6′-hydroxy-SRBs to form SRBs. The *srrO* recombinant converted 6′-deoxo-SRB1 **1** to SRB1 in a time-dependent manner, whereas the control recombinant harboring empty vector pHSA81 was unable to oxidize **1** (Figure 5). In addition, biosynthetic intermediate 6′-deoxo-6′-hydroxy-SRB1 could not be converted to SRB1 in the control recombinant *S. lividans* TK64/pHSA81 (data not shown). Hence, two-stage oxidation is enzymatically catalyzed by SrrO in SRB biosynthesis. SrrO could oxidize the C-6′ methylene group on not only natural (1’*R*)-isomers **1a** but unnatural (1’*S*)-isomers **1b** (Figure 6). This finding indicated that C-1’ stereochemistry of SRBs is strictly controlled at an earlier biosynthetic step of SrrO, possibly by NAD-dependent dehydrogenase SrrG.

**Figure 7:**
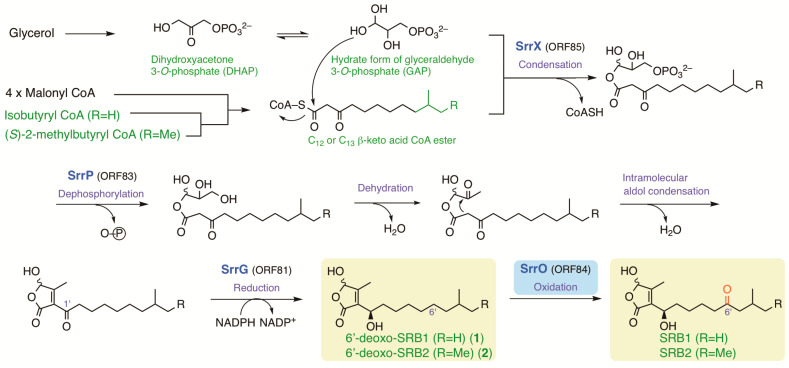
The possible biosynthetic pathway of SRBs in *S. rochei*. Me; methyl.

Several possible genes for SRB biosynthesis and antibiotic regulation were found around *srrX* (*orf85*) on pSLA2-L; the NAD-dependent dehydrogenase gene *srrG* (*orf81*), the phosphatase gene *srrP* (*orf83*), the P450 monooxygenase gene *srrO* (*orf84*), and the thioesterase gene *srrH* (*orf86*), together with repressor genes *srrA* (*orf82*) and *srrB* (*orf79*). The possible biosynthetic pathway of SRBs were shown in Figure 7 based on the biosynthetic pathways for other signaling molecules, A-factor in *S. griseus* [8] and virginia butanolides in *S. virginiae* [43], and antifungal butenolide gladiofungin in *Burkholderia gladioli* HKI0739 [44]. Medium-chain (C_12_ or C_13_) β-keto acid was derived through fatty acid biosynthesis pathway; four units of malonyl CoA are condensed with either an isobutyryl CoA unit for SRB1 or a 2-methylbutyryl CoA unit for SRB2. These β-keto acids are condensed with a C_3_ unit (a hydrate form of glyceraldehyde 3-phosphate) by SrrX, then followed by spontaneous dephosphorylation, dihydroxylation, and intramolecular aldol condensation to generate the butenolide skeleton. The C-1’ ketone moiety in butenolide intermediate will be reduced by dehydrogenase SrrG to synthesize 6′-deoxo-SRBs, which accept two-stage oxidation by SrrO to form SRBs.

*Streptomyces* signaling molecules have a crucial role to induce secondary metabolite biosynthesis. Many *Streptomyces* strains have more than 30 secondary metabolites biosynthetic gene clusters in their genome; however, many of them (around 80–90%) are silent in normal culture conditions. In *S. rochei*, 40 biosynthetic gene clusters (35 in the chromosome and 5 in pSLA2-L) are found; however, only six compounds are detected [17,45]. Although we do not yet know the reason why many of them are silent or poorly expressed, a lack of specific signaling molecules is one of a plausible possibility [46,47]. *Streptomyces* strains generally have multiple signaling-molecule receptor genes, suggesting that they may have a potential to recognize heterologous signaling molecules. Thus, signaling molecules may contribute as “genetic engineering-free” genome mining tools to act as communication signals between actinomycetes, between different bacteria, and/or between interkingdom.

## 5. Conclusions

We here investigated the role of a P450 monooxygenase gene *srrO* in SRB biosynthesis. Two signaling molecules in the *srrO*-disruptant KA54 were determined to be 6′-deoxo-SRB1 and 6′-deoxo-SRB2 by ESI-MS, NMR, and HPLC analyses from 30 L culture extract. A ligand affinity of 6′-deoxo-SRB1 to SrrA was shown to be 100-fold less than that of SRB1. We further performed bioconversion of the synthetic 6′-deoxo-SRB1 in the *S. lividans* recombinant carrying SrrO-expression plasmid. Substrate 6′-deoxo-SRB1 was converted through 6′-deoxo-6′-hydroxy-SRB1 to SRB1 in a time-dependent manner. Thus, these results clearly indicated that SrrO catalyzes the C-6′ oxidation at a final step in SRB biosynthesis.

## Supplementary Materials

The followings are available online at: https://www.mdpi.com/2218-273X/10/9/1237/s1. Table S1: Bacterial strains, plasmids, and oligonucleotides used in this study; Figure S1: Chemical structures of lankamycin, lankacidin C, lankacidinol A, and lankacidinol; Figure S2: Gene disruption of *srrO* (*orf84*); Figure S3: Overexpression of the SrrO protein; Figure S4: ^1^H-NMR of 6-hydroxyhexyl *p*-toluenesulfonate (**4**); Figure S5: ^13^C-NMR of 6-hydroxyhexyl *p*-toluenesulfonate (**4**); Figure S6: ^1^H-NMR of 6-((tetrahydro-*2H*-pyran-2-yl)oxy)hexyl *p*-toluenesulfonate (**5**); Figure S7: ^13^C-NMR of 6-((tetrahydro-*2H*-pyran-2-yl)oxy)hexyl *p*-toluenesulfonate (**5**); Figure S8: ^1^H-NMR of 2-((8-methylnonyl)oxy)tetrahydro-*2H*-pyran (**6**); Figure S9: ^13^C-NMR of 2-((8-methylnonyl)oxy)tetrahydro-*2H*-pyran (**6**); Figure S10: ^1^H-NMR of 8-menthylnonan-1-ol (**7**); Figure S11: ^13^C-NMR of 8-menthylnonan-1-ol (**7**); Figure S12: ^1^H-NMR of 8-menthylnonanal (**8**); Figure S13: ^13^C-NMR of 8-menthylnonanal (**8**); Figure S14: ^1^H-NMR of compound **10a**; Figure S15: ^13^C-NMR of compound **10a**; Figure S16: ^1^H-NMR of compound **10b**; Figure S17: ^13^C-NMR of compound **10b**; Figure S18: ^1^H-NMR of 6′-deoxo-SRB1a (**1a**); Figure S19: ^13^C-NMR of 6′-deoxo-SRB1a (**1a**); Figure S20: ^1^H-NMR of 6′-deoxo-SRB1b (**1b**); Figure S21: ^13^C-NMR of 6′-deoxo-SRB1b (**1b**); Figure S22: ^1^H-NMR of 2-(((*S*)-8-methyldecyl)oxy)tetrahydro-*2H*-pyran (**11**); Figure S23: ^13^C-NMR of 2-(((*S*)-8-methyldecyl)oxy)tetrahydro-*2H*-pyran (**11**); Figure S24: ^1^H-NMR of (*S*)-8-methyldecan-1-ol (**12**); Figure S25: ^13^C-NMR of (*S*)-8-methyldecan-1-ol (**12**); Figure S26: ^1^H-NMR of (*S*)-8-methyldecanal (**13**); Figure S27: ^13^C-NMR of (*S*)-8-methyldecanal (**13**); Figure S28: ^1^H-NMR of compound **14a**; Figure S29: ^13^C-NMR of compound **14a**; Figure S30: ^1^H-NMR of compound **14b**; Figure S31: ^13^C-NMR of compound **14b**; Figure S32: ^1^H-NMR of 6′-deoxo-SRB2a (**2a**); Figure S33: ^13^C-NMR of 6′-deoxo-SRB2a (**2a**); Figure S34: ^1^H-NMR of 6′-deoxo-SRB2b (**2b**); Figure S35: ^13^C-NMR of 6′-deoxo-SRB2b (**2b**).
