# Supplementary material for: Functional Analysis of P450 Monooxygenase SrrO in the Biosynthesis of Butenolide-Type Signaling Molecules in Streptomyces rochei
Source: Biomolecules. 2020 Aug 25;10(9):1237. doi: 10.3390/biom10091237 (PMC7564063; doi:10.3390/biom10091237)
Supplement: Supplementary file 1 [file biomolecules-10-01237-s001.pdf]

Supplementary Materials

Functional Analysis of P450 Monooxygenase SrrO in the Biosynthesis of Butenolide-type Signaling Molecules in *Streptomyces rochei*

Aiko Teshima[1], Nozomi Hadae[1], Naoto Tsuda[1], and Kenji Arakawa[1,2,*]

[1] Department of Molecular Biotechnology, Graduate School of Advanced Sciences of Matter, Hiroshima University, 1-3-1 Kagamiyama, Higashi-Hiroshima, Hiroshima 739-8530, Japan

[2] Unit of Biotechnology, Graduate School of Integrated Sciences for Life, Hiroshima University, 1-3-1 Kagamiyama, Higashi-Hiroshima, Hiroshima 739-8530, Japan

* Correspondence: karakawa@hiroshima-u.ac.jp; Tel. & Fax: +81-82-424-7767

Contents

Table S1: Bacterial strains, plasmids, and oligonucleotides used in this study
Figure S1: Chemical structures of lankamycin, lankacidin C, lankacidinol A, and lankacidinol
Figure S2: Gene disruption of *srrO* (*orf84*)
Figure S3: Overexpression of the SrrO protein
Figure S4: ^{1}H-NMR of 6-hydroxyhexyl *p*-toluenesulfonate (**4**)
Figure S5: ^{13}C-NMR of 6-hydroxyhexyl *p*-toluenesulfonate (**4**)
Figure S6: ^{1}H-NMR of 6-((tetrahydro-*2H*-pyran-2-yl)oxy)hexyl *p*-toluenesulfonate (**5**)
Figure S7: ^{13}C-NMR of 6-((tetrahydro-*2H*-pyran-2-yl)oxy)hexyl *p*-toluenesulfonate (**5**)
Figure S8: ^{1}H-NMR of 2-((8-methylnonyl)oxy)tetrahydro-*2H*-pyran (**6**)
Figure S9: ^{13}C-NMR of 2-((8-methylnonyl)oxy)tetrahydro-*2H*-pyran (**6**)
Figure S10: ^{1}H-NMR of 8-menthylnonan-1-ol (**7**)
Figure S11: ^{13}C-NMR of 8-menthylnonan-1-ol (**7**)
Figure S12: ^{1}H-NMR of 8-menthylnonanal (**8**)
Figure S13: ^{13}C-NMR of 8-menthylnonanal (**8**)
Figure S14: ^{1}H-NMR of compound **10a**
Figure S15: ^{13}C-NMR of compound **10a**
Figure S16: ^{1}H-NMR of compound **10b**
Figure S17: ^{13}C-NMR of compound **10b**
Figure S18: ^{1}H-NMR of 6'-deoxo-SRB1a (**1a**)
Figure S19: ^{13}C-NMR of 6'-deoxo-SRB1a (**1a**)
Figure S20: ^{1}H-NMR of 6'-deoxo-SRB1b (**1b**)
Figure S21: ^{13}C-NMR of 6'-deoxo-SRB1b (**1b**)
Figure S22: ^{1}H-NMR of 2-(((*S*)-8-methyldecyl)oxy)tetrahydro-*2H*-pyran (**11**)
Figure S23: ^{13}C-NMR of 2-(((*S*)-8-methyldecyl)oxy)tetrahydro-*2H*-pyran (**11**)
Figure S24: ^{1}H-NMR of (*S*)-8-methyldecan-1-ol (**12**)
Figure S25: ^{13}C-NMR of (*S*)-8-methyldecan-1-ol (**12**)
Figure S26: ^{1}H-NMR of (*S*)-8-methyldecanal (**13**)
Figure S27: ^{13}C-NMR of (*S*)-8-methyldecanal (**13**)
Figure S28: ^{1}H-NMR of compound **14a**
Figure S29: ^{13}C-NMR of compound **14a**
Figure S30: ^{1}H-NMR of compound **14b**
Figure S31: ^{13}C-NMR of compound **14b**
Figure S32: ^{1}H-NMR of 6'-deoxo-SRB2a (**2a**)
Figure S33: ^{13}C-NMR of 6'-deoxo-SRB2a (**2a**)
Figure S34: ^{1}H-NMR of 6'-deoxo-SRB2b (**2b**)
Figure S35: ^{13}C-NMR of 6'-deoxo-SRB2b (**2b**)

Table S1. Bacterial strains, plasmids, and oligonucleotides used in this study

Strains/plasmids/oligonucleotides	Properties/product	Source/ref.[*1]
Strains		
S. rochei		
7434AN4	Wild type (pSLA2-L,M,S)	[12]
51252	pSLA2-L	[12]
KA20	*ΔsrrXΔsrrB* in strain 51252	[21]
KA54	*ΔsrrO* in strain 51252	This study
S. lividans		
TK64	*pro-2*, *str-6*	[30]
TK64/pNTT01	Strain TK64 with plasmid pNTT01, *tsr*, $(His)_6$-tagged *srrO*	This study
TK64/pHSA81	Strain TK64 with plasmid pHSA81, *tsr*	[38]
E. coli		
XL1-Blue	*recA1 endA1 gyrA96 thi-1 hsdR17 supE44 relA1 lac* [F'*proAB lacI*^{q}*ZΔM15* Tn*10* (*tet*)]	Stratagene
Plasmids		
SuperCos-1	Cosmid vector, *amp*, *kan*	Stratagene
cosmid C7	41.3-kb pSLA2-L DNA (nt 143,101-184,445) cloned into SuperCos-1 at *Bam*HI site	[17]
pRES18	*E. coli*-*Streptomyces* shuttle vector, *amp*, *tsr*, *lacZ*-α	[33]
Litmus 28i	*E. coli* cloning vector, *amp*, *lacZ*-α	New England Biolabs
pKAR3041	3.8-kb *Eco*RI-*Stu*I fragment containing *srrO* in Litmus 28i	[19]
pKAR3043	1.0-kb *ClaI* fragment of aac(3)IV gene carrying apramycin resistance into ClaI site of pKAR304	This study
pKAR3044	2.3-kb *Bam*HI fragment carrying *ΔlkmF* in pRES18	This study
pHSA81	Constitutive expression vector in *Streptomyces*, *tsr*	M. Kobayashi
pKAR3063H	Constitutive expression vector in *Streptomyces*, *tsr*, N-terminal $(His)_6$-tag	[23]
pNTT01	1.2 kb *Nde*I-*Hin*dIII PCR fragment carrying *srrO* cloned into pKAR3063H	This study
Designed oligonucleotides		
KAR-APR05	5'-GCGAATTCGCATGCATCGATACAGAATGAT-3'	This study
KAR-APR06	5'-TGTAAGCTTATCGATGCATGCACGTGTTGC-3'	This study
NT-srrO-OE-F	5'-ATACATATGCTTCGTCAGGAAGCGCCCTA-3'	This study
NT-srrO-OE-R	5'-TTAAAGCTTCATGCCGCGGCTCCGGGCAC-3'	This study

*1; Reference numbers are identical with those indicated in main text.

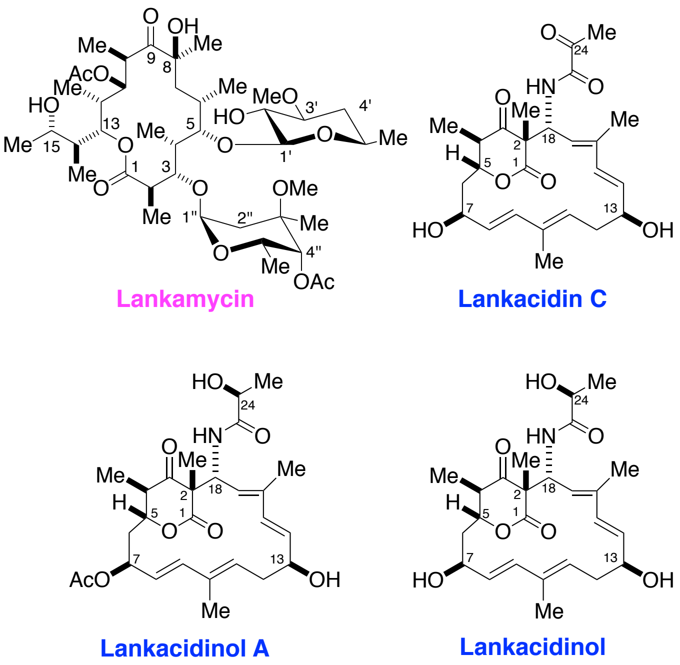

Figure S1: Chemical structures of lankamycin, lankacidin C, lankacidinol A, and lankacidinol. Me, methyl; Ac, acetyl.

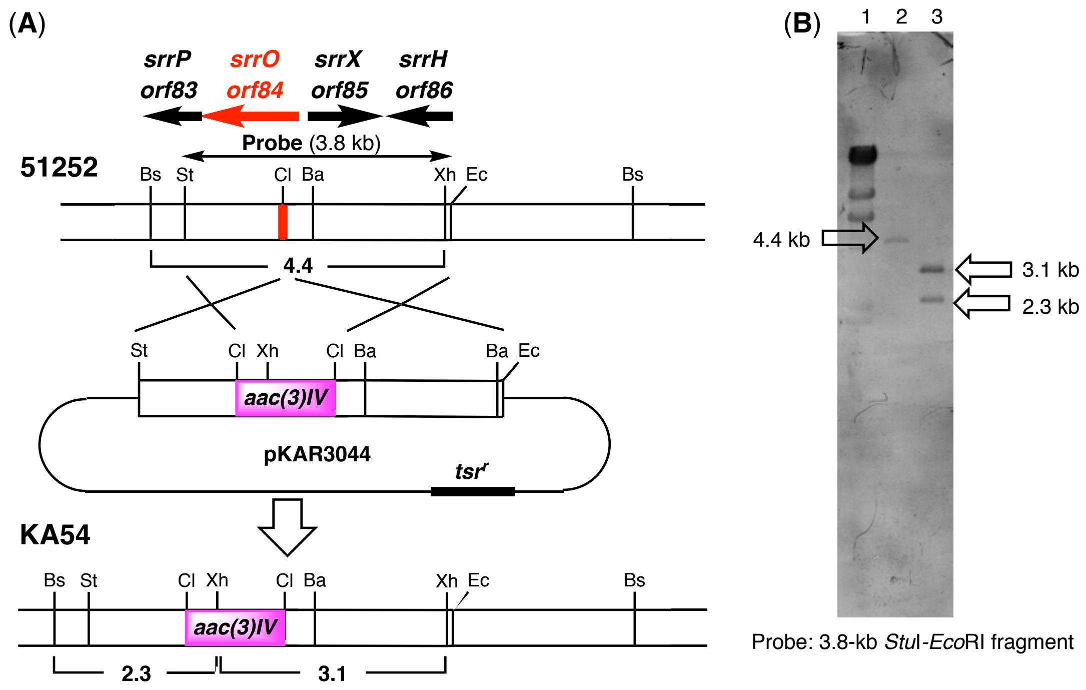

Figure S2: Gene disruption of *srrO* (*orf84*). (**A**) Construction of the *srrO* mutant KA54. St, *Stu*I; Ec, *Eco*RI; Bs, *Bsp*EI; Cl, *Cla*I; Xh, *Xho*I. *aac(3)IV*, apramycin resistance gene cassette; *tsr*^{r}, thiostrepton resistance gene cassette. (**B**) Southern blot analysis. Lane 1, λ/*Hin*dIII; lane 2, 51252 (parent)/*Bsp*EI-*Xho*I; lane 3, KA54 (Δ*srrO*)/*Bsp*EI-*Xho*I.

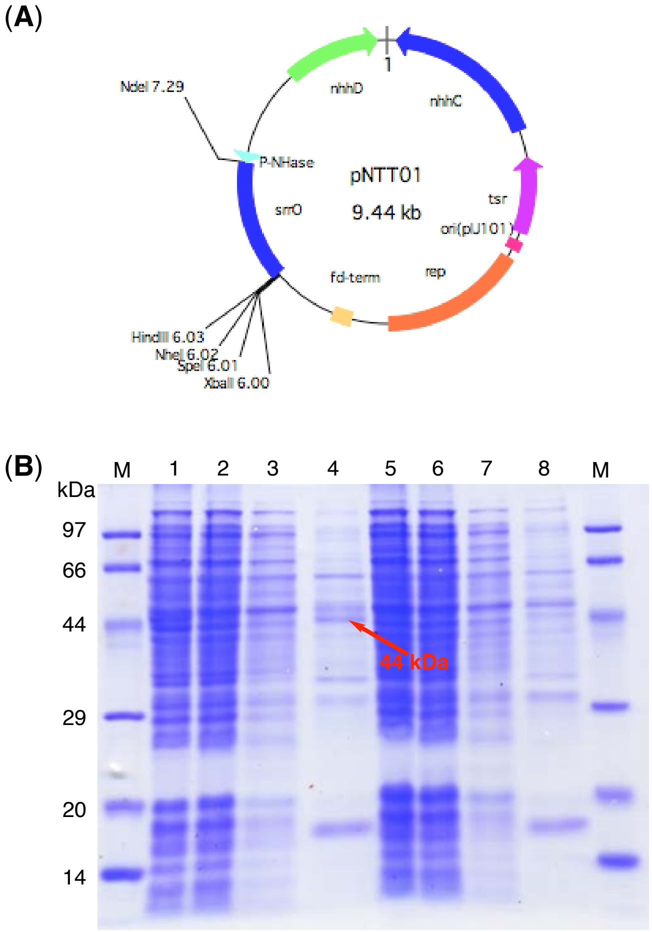

Figure S3: Overexpression of the SrrO protein. (**A**) Physical map of the SrrO overexpression plasmid pNTT01, a vector part of whicn was pHSA81 derivative (Profs. Y. Hashimoto and M. Kobayashi, personal communication). (**B**) SDS-PAGE of the recombinant SrrO protein expressed in *S. lividans* TK64. Lane M, molecular size marker; lane 1, cell-free supernatant of the *S. lividans* TK64/pNTT01 recombinant (+ SrrO); lane 2, cell-free supernatant of the *S. lividans* TK64/pNTT01 recombinant; lane 3, wash fraction of the *S. lividans* TK64/pNTT01 recombinant; lane 4, elution fraction of the *S. lividans* TK64/pNTT01 recombinant; lane 5, cell-free supernatant of the *S. lividans* TK64/pHSA81 recombinant (control); lane 5, flow-through fraction of the *S. lividans* TK64/pHSA81 recombinant; lane 5, wash fraction of the *S. lividans* TK64/pHSA81 recombinant; lane 5, elution fraction of the *S. lividans* TK64/pHSA81 recombinant. Purification of $(His)_6$-tagged protein was performed according to the manufacture's protocol.

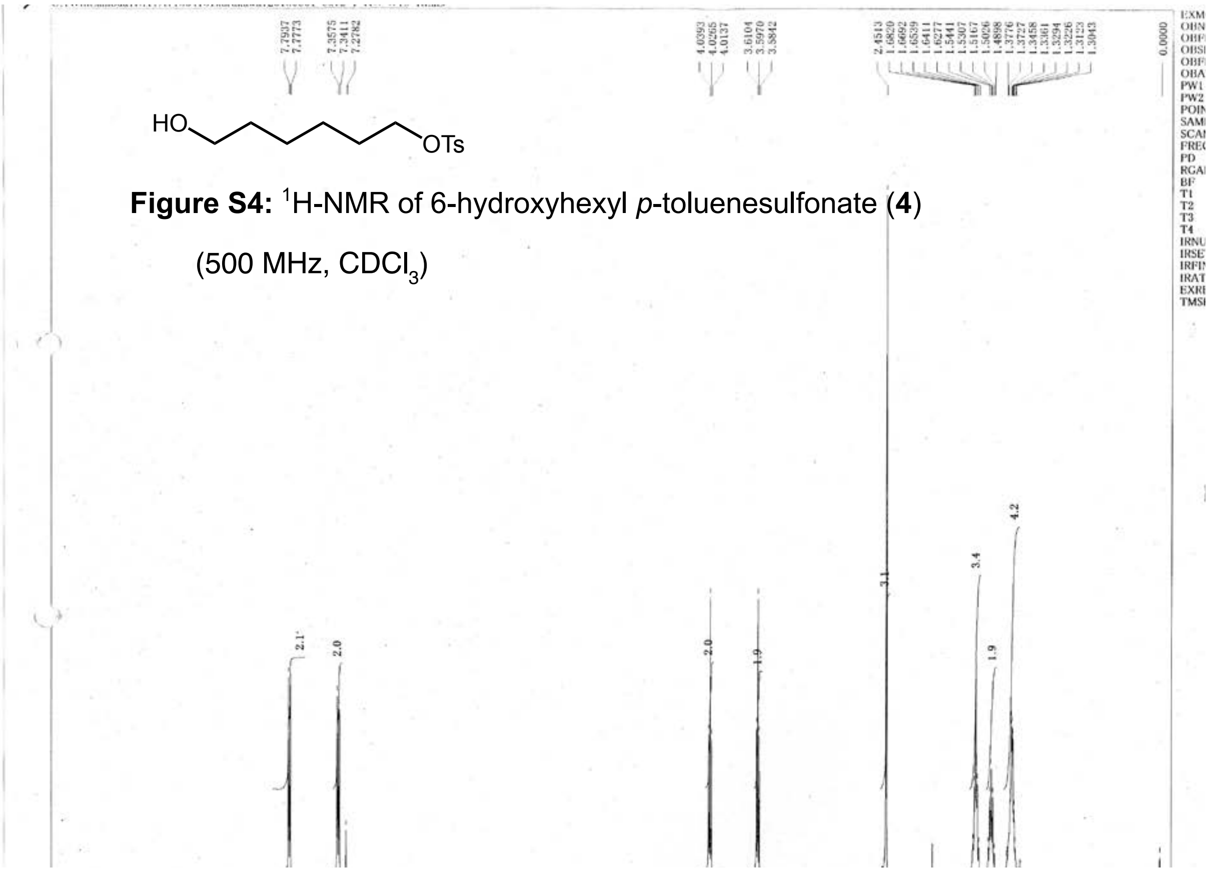

Figure S4: ^{1}H-NMR of 6-hydroxyhexyl *p*-toluenesulfonate (**4**)

(500 MHz, $CDCl_3$)

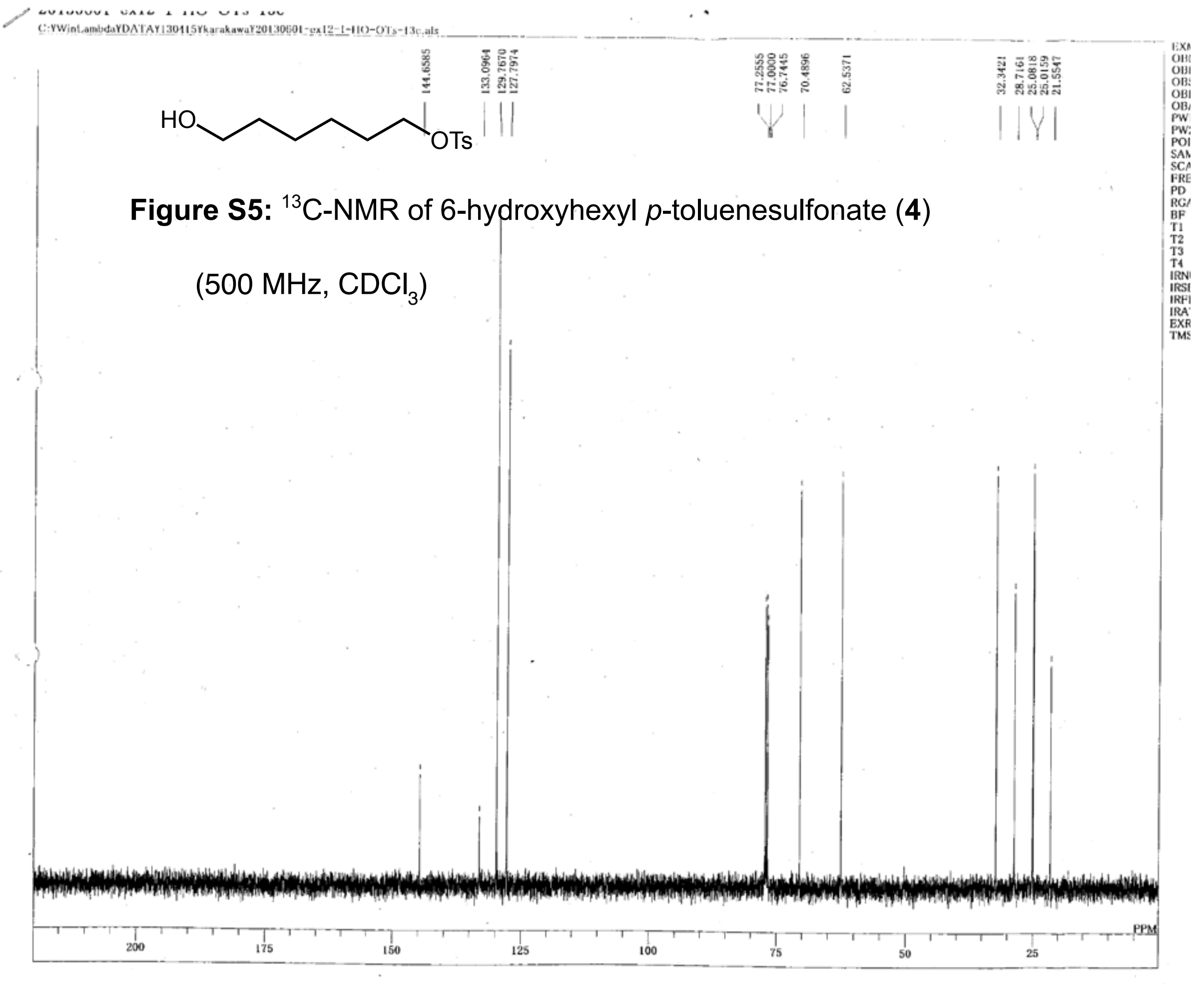

Figure S5: ^{13}C-NMR of 6-hydroxyhexyl *p*-toluenesulfonate (**4**)

(500 MHz, $CDCl_3$)

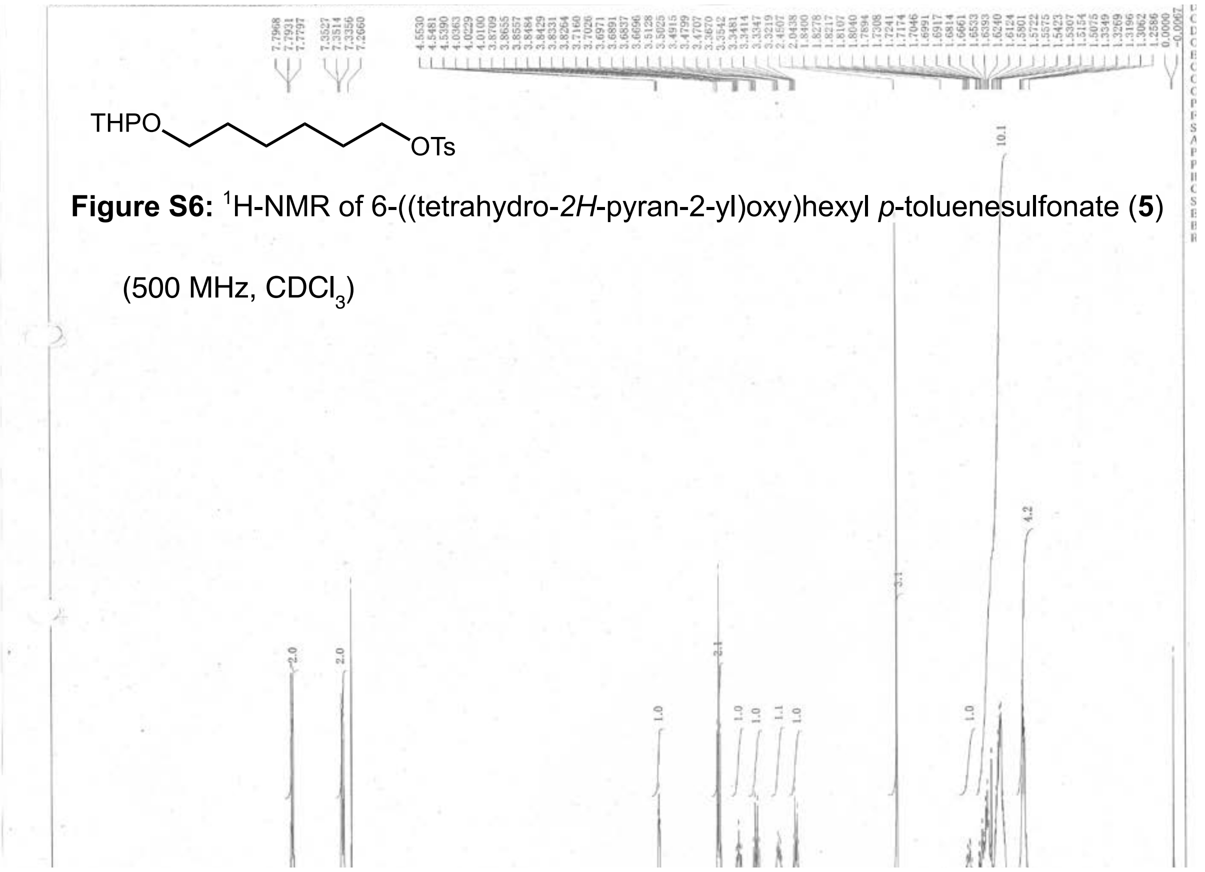

Figure S6: ¹H-NMR of 6-((tetrahydro-*2H*-pyran-2-yl)oxy)hexyl *p*-toluenesulfonate (**5**)

(500 MHz, $CDCl_3$)

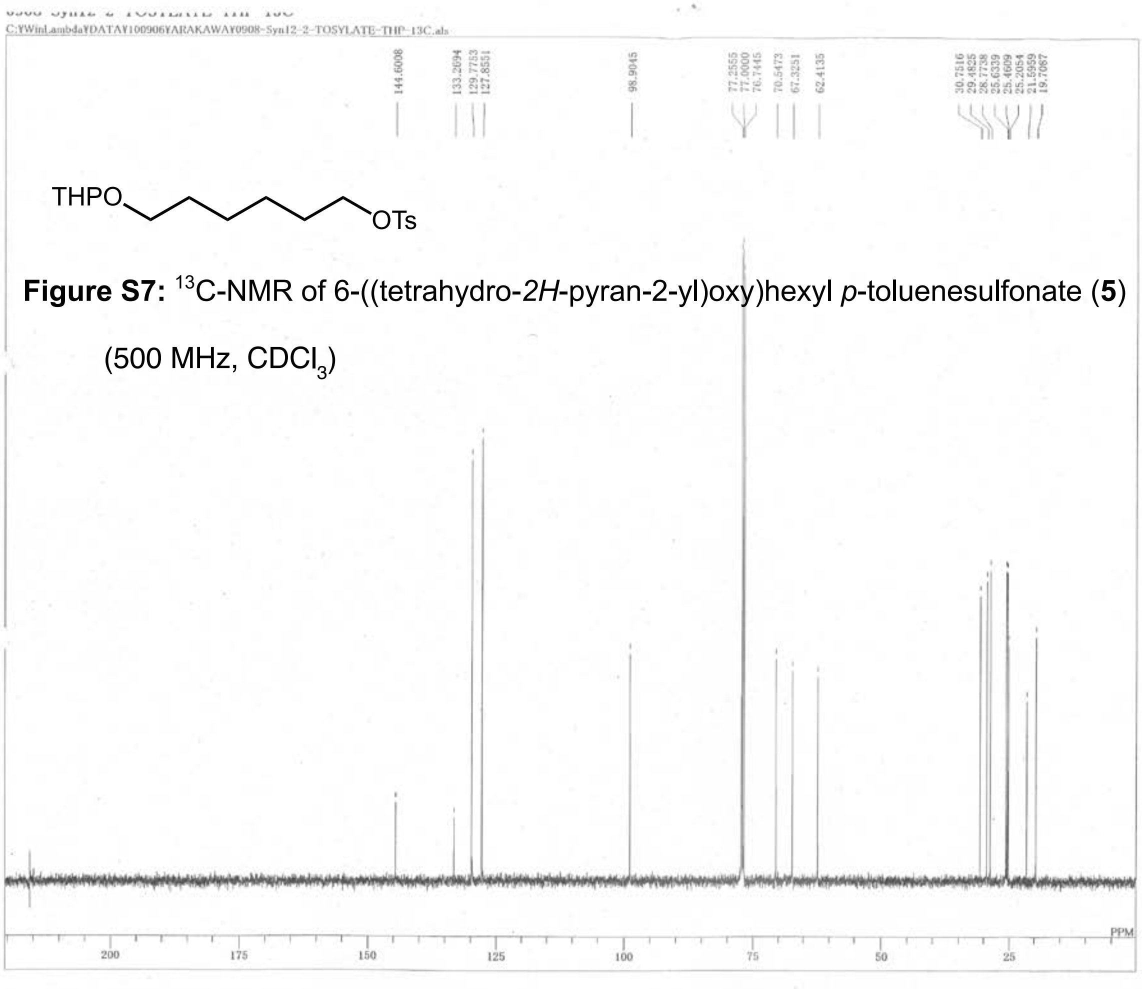

Figure S7: ¹³C-NMR of 6-((tetrahydro-*2H*-pyran-2-yl)oxy)hexyl *p*-toluenesulfonate (**5**)

(500 MHz, $CDCl_3$)

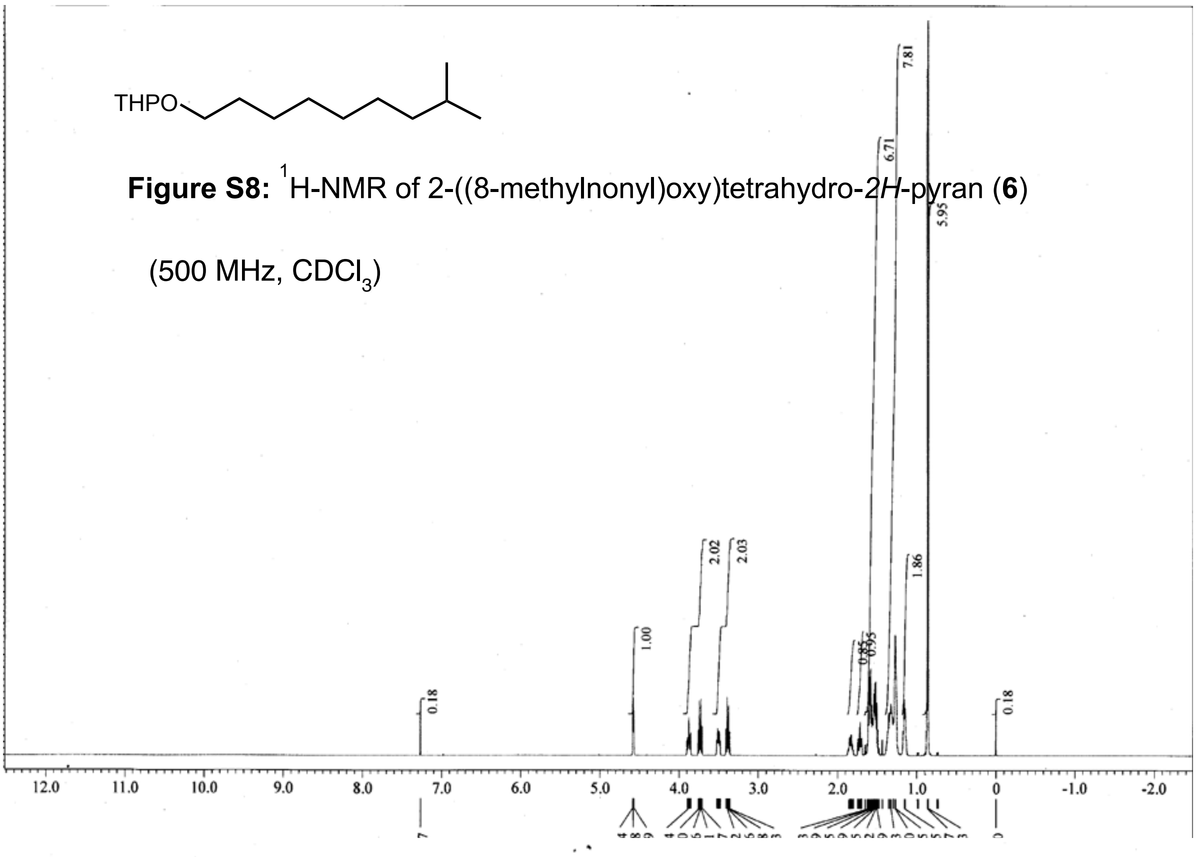

Figure S8: ^{1}H-NMR of 2-((8-methylnonyl)oxy)tetrahydro-*2H*-pyran (**6**)

(500 MHz, $CDCl_3$)

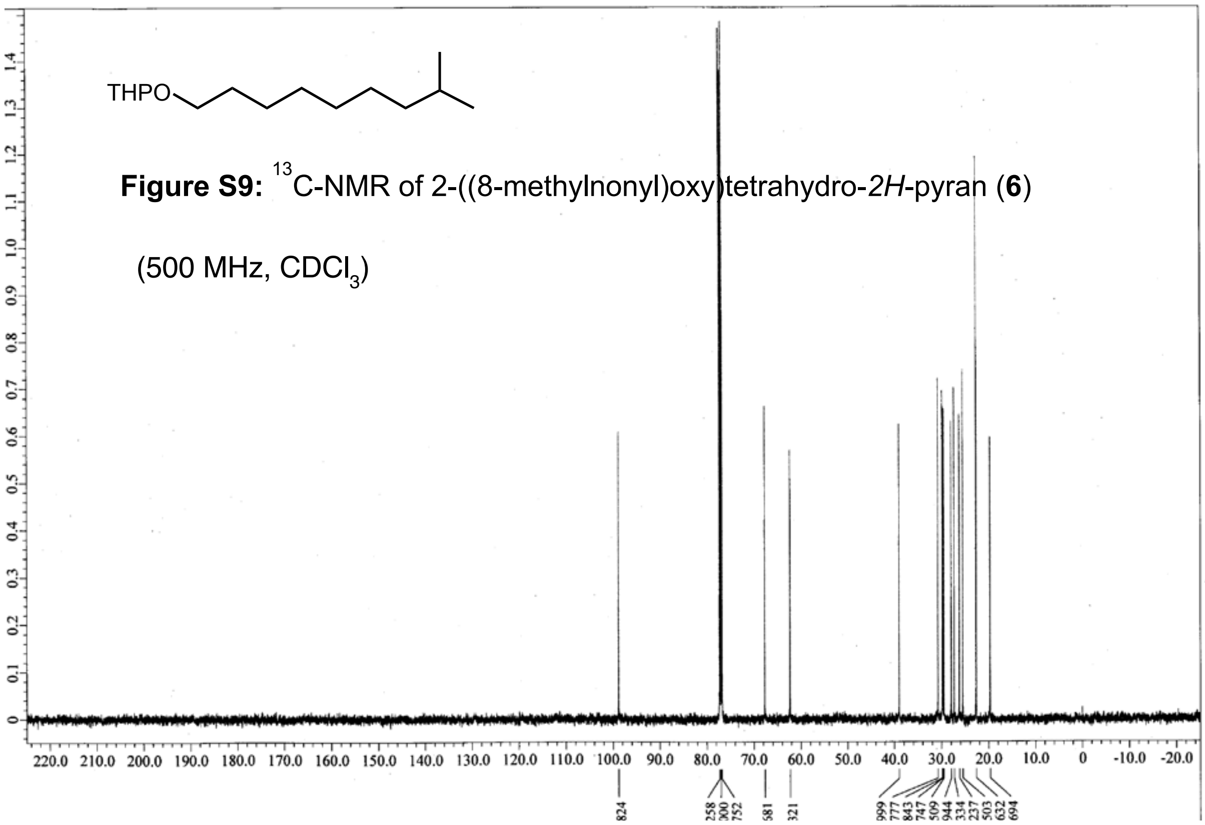

Figure S9: ^{13}C-NMR of 2-((8-methylnonyl)oxy)tetrahydro-*2H*-pyran (**6**)

(500 MHz, $CDCl_3$)

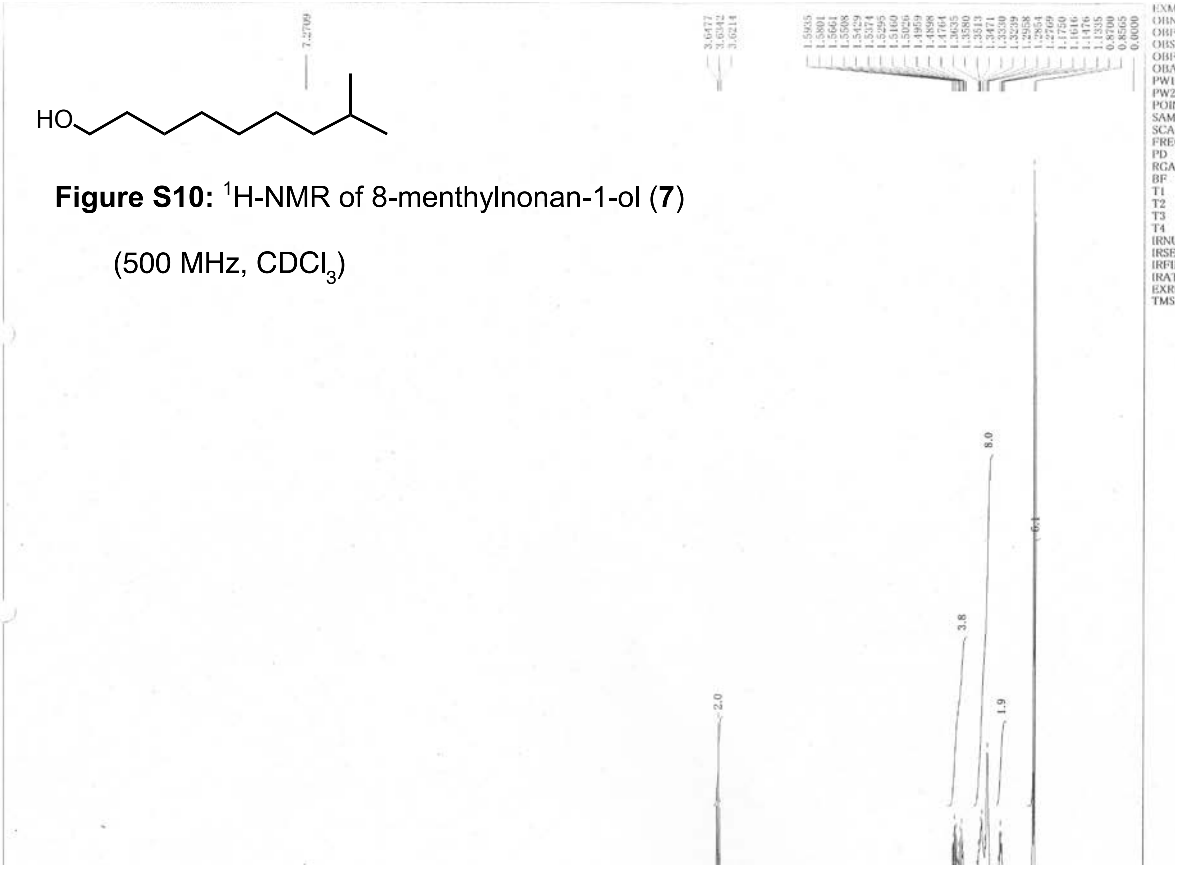

Figure S10: ^{1}H-NMR of 8-menthylnonan-1-ol (**7**)

(500 MHz, $CDCl_3$)

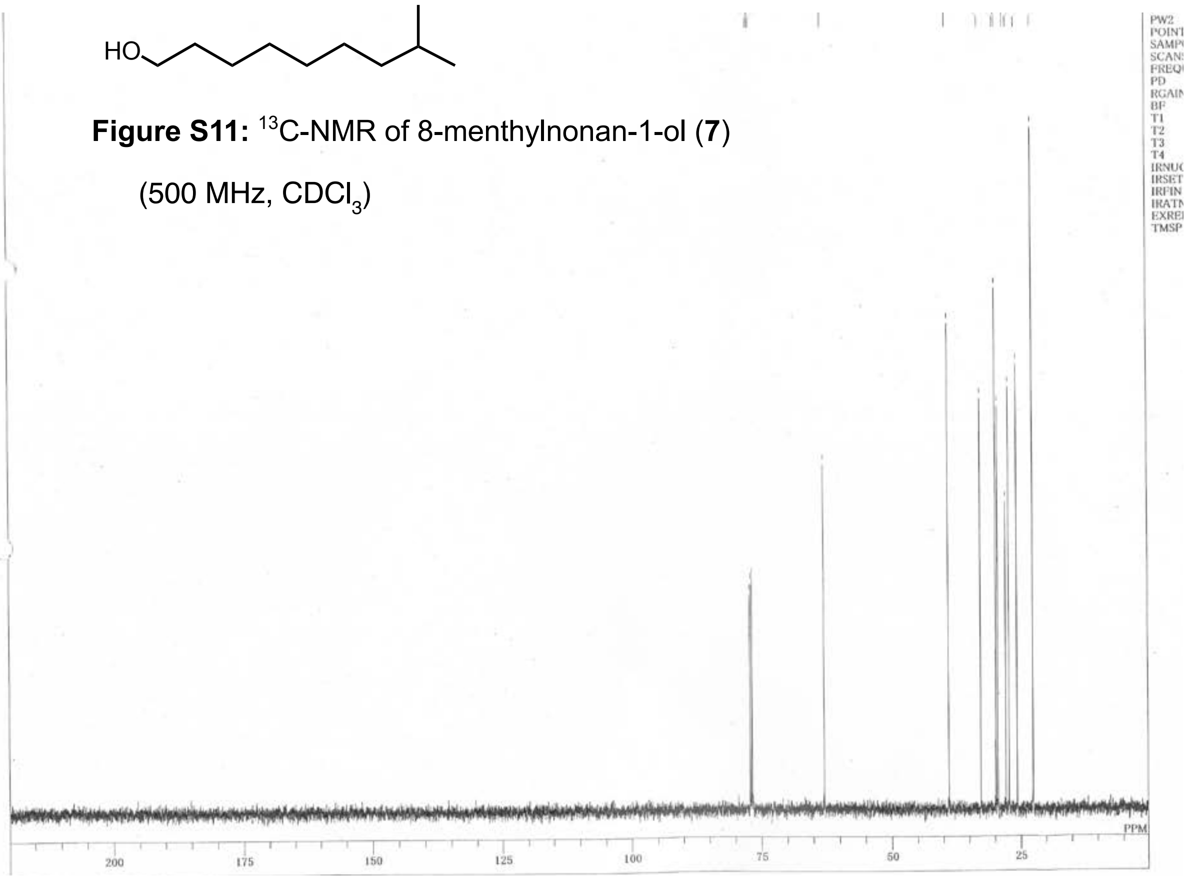

Figure S11: ^{13}C-NMR of 8-menthylnonan-1-ol (**7**)

(500 MHz, $CDCl_3$)

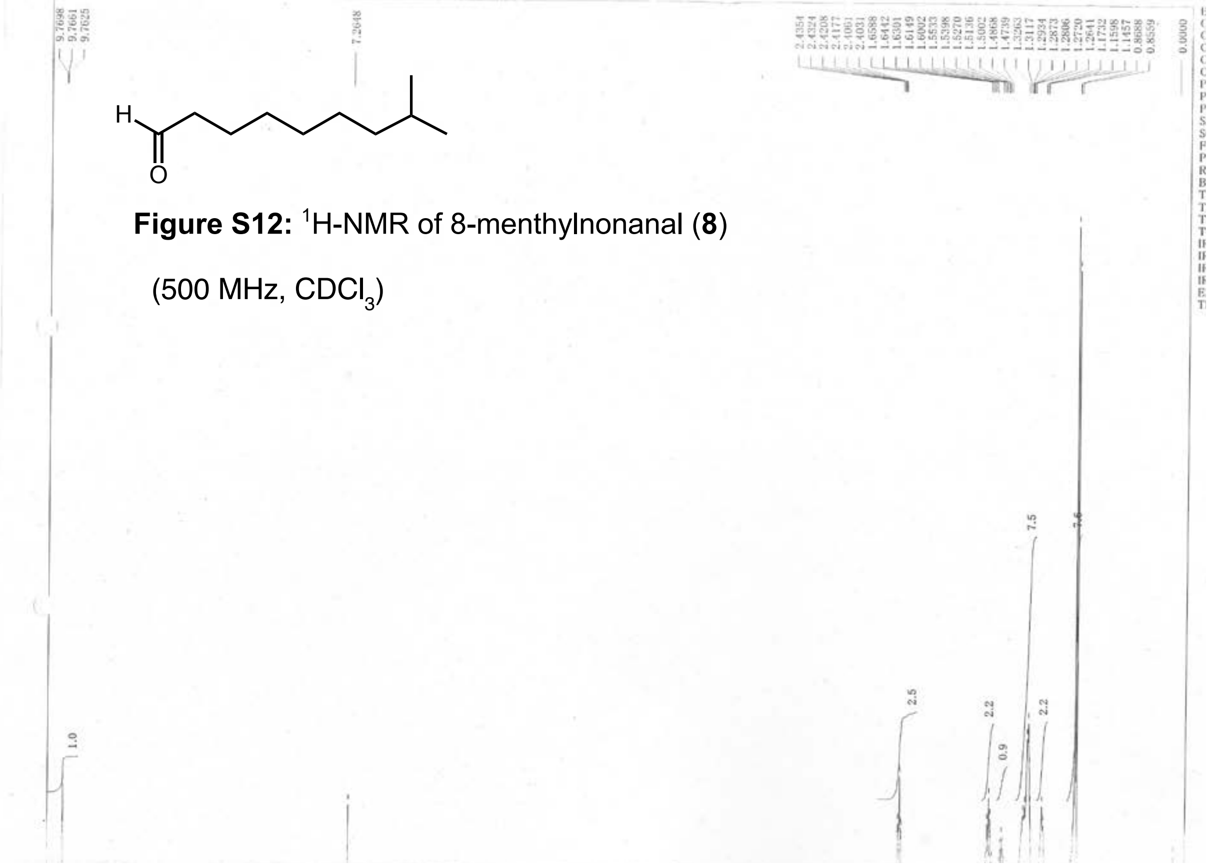

Figure S12: ^{1}H-NMR of 8-menthylnonanal (**8**)

(500 MHz, $CDCl_3$)

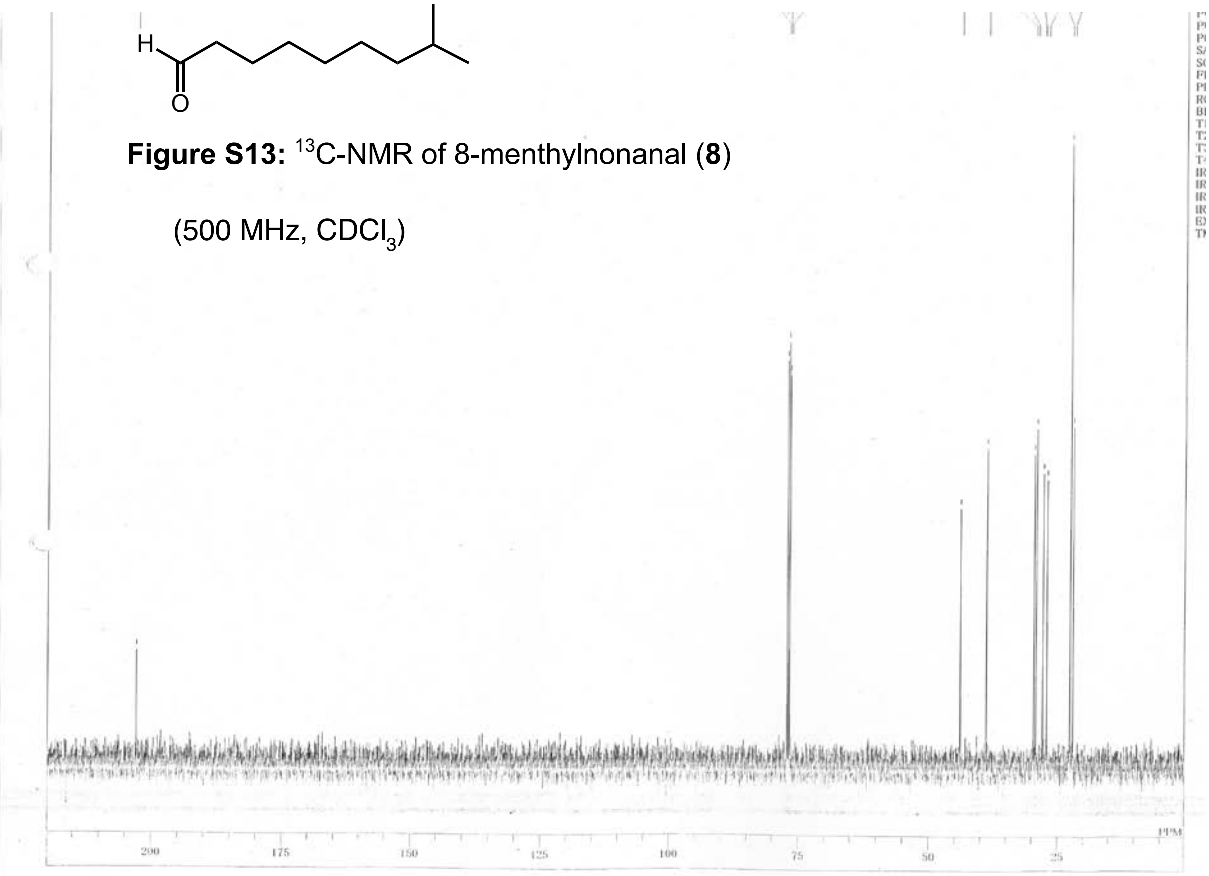

Figure S13: ^{13}C-NMR of 8-menthylnonanal (**8**)

(500 MHz, $CDCl_3$)

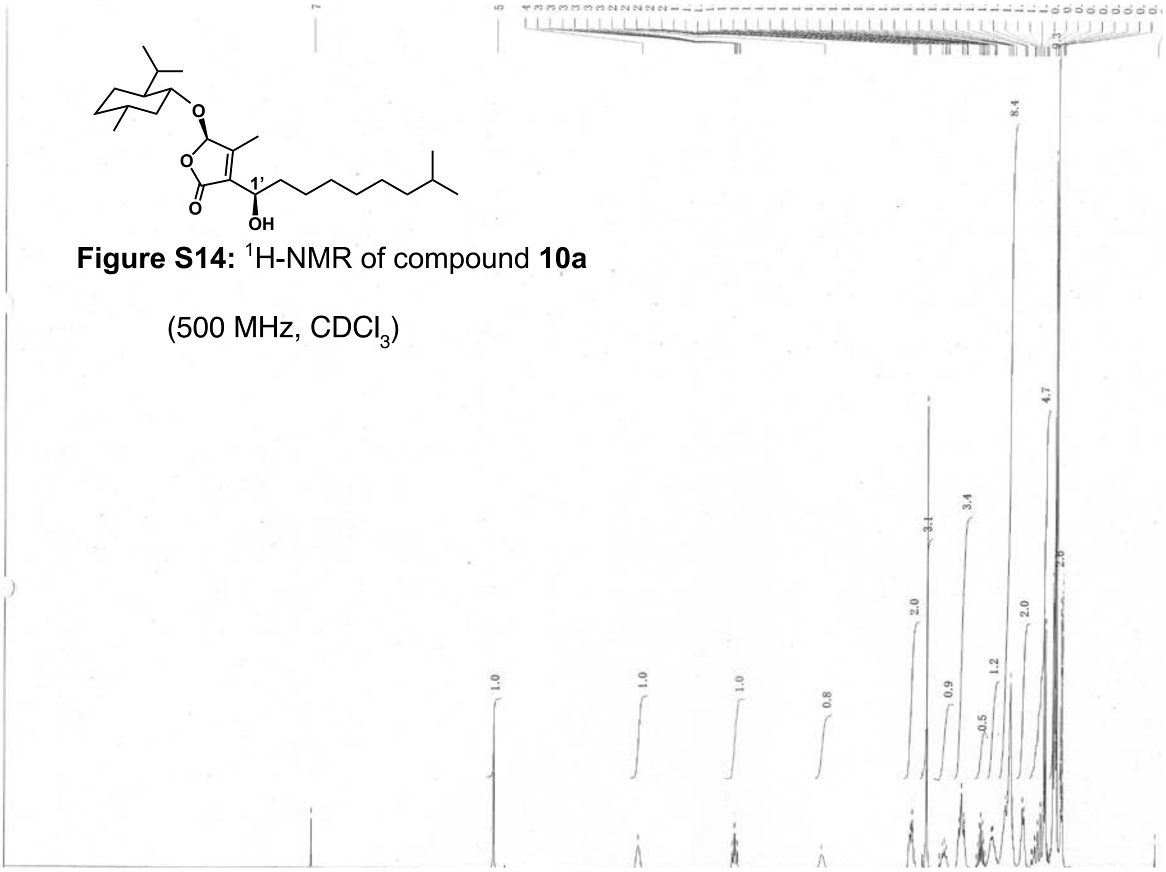

Figure S14: ^{1}H-NMR of compound **10a**

(500 MHz, $CDCl_3$)

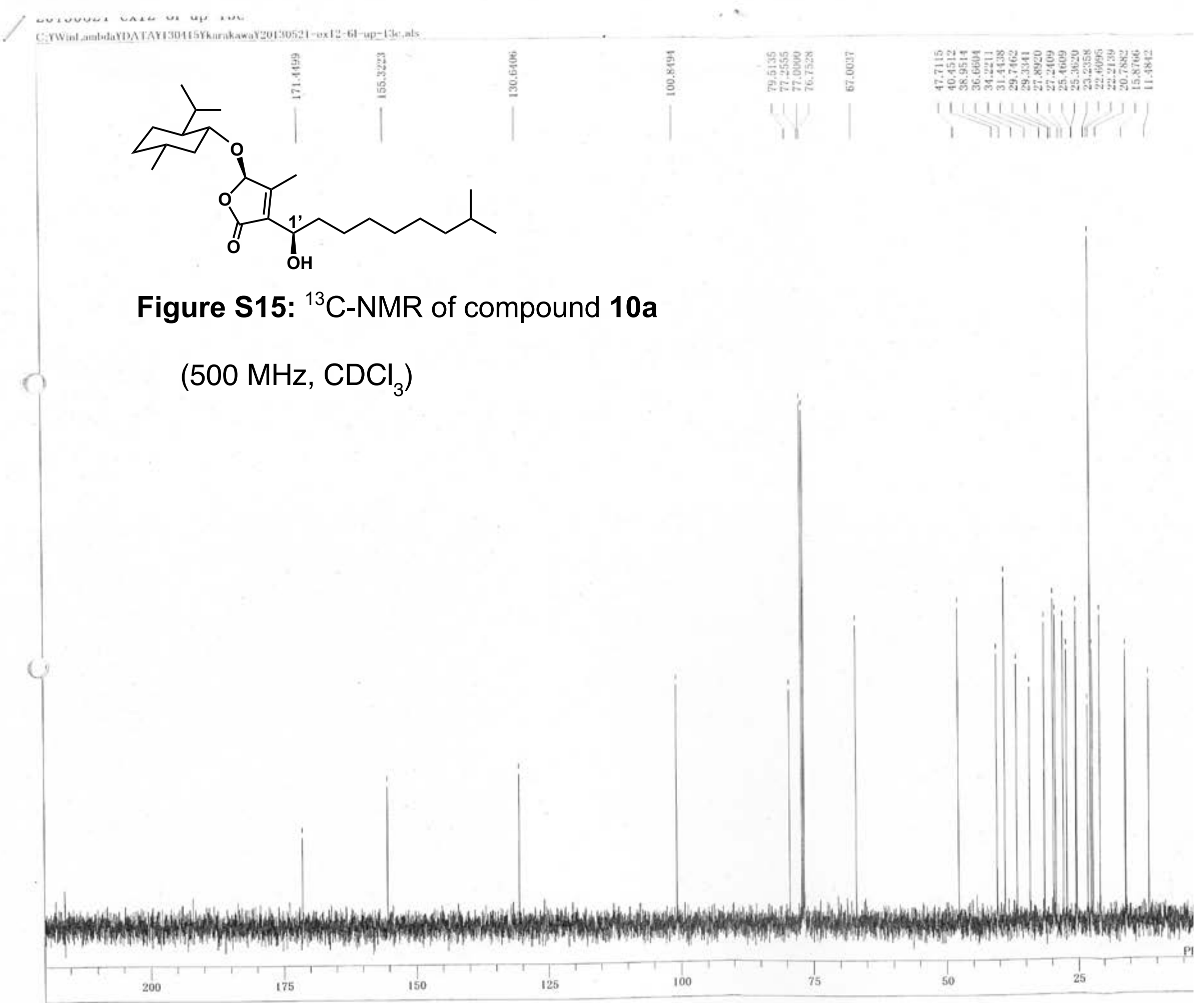

Figure S15: ^{13}C-NMR of compound **10a**

(500 MHz, $CDCl_3$)

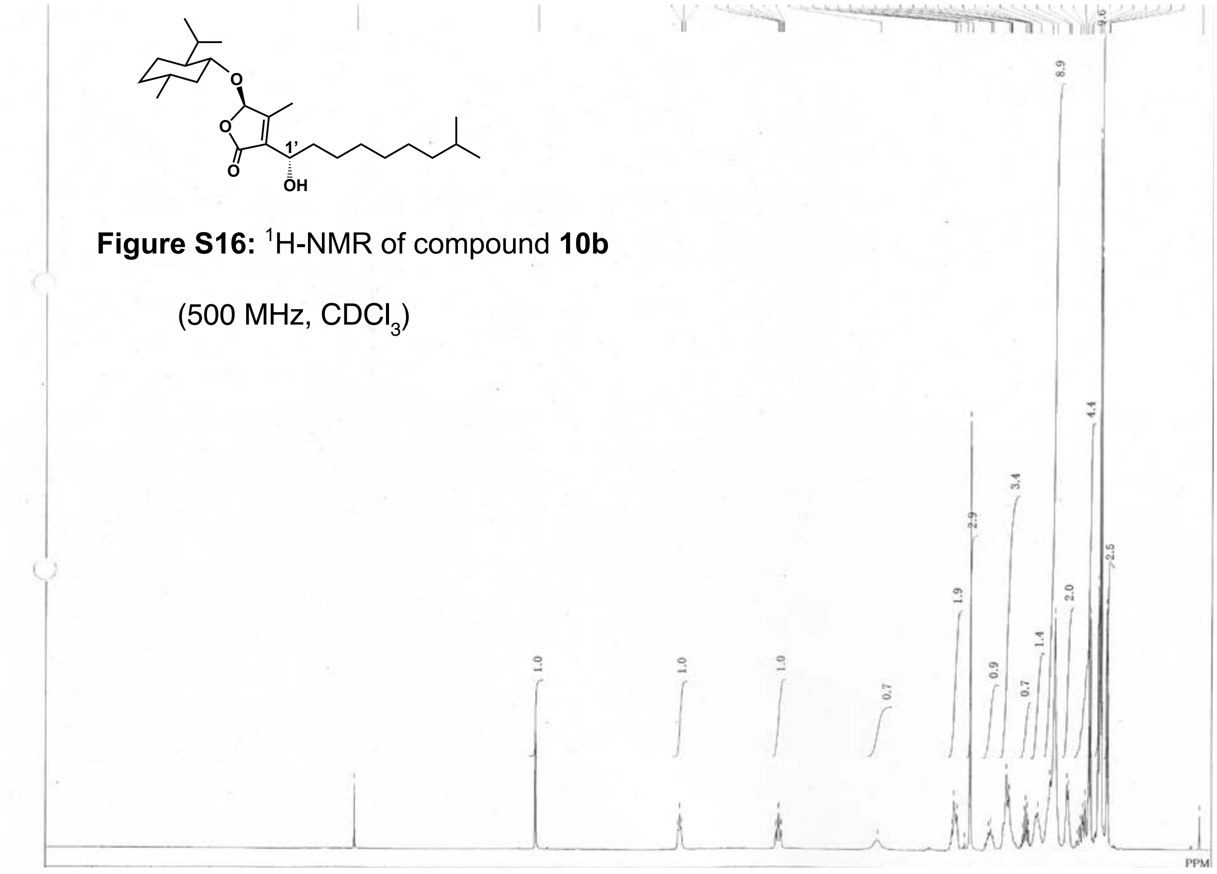

Figure S16: ^{1}H-NMR of compound **10b**

(500 MHz, $CDCl_3$)

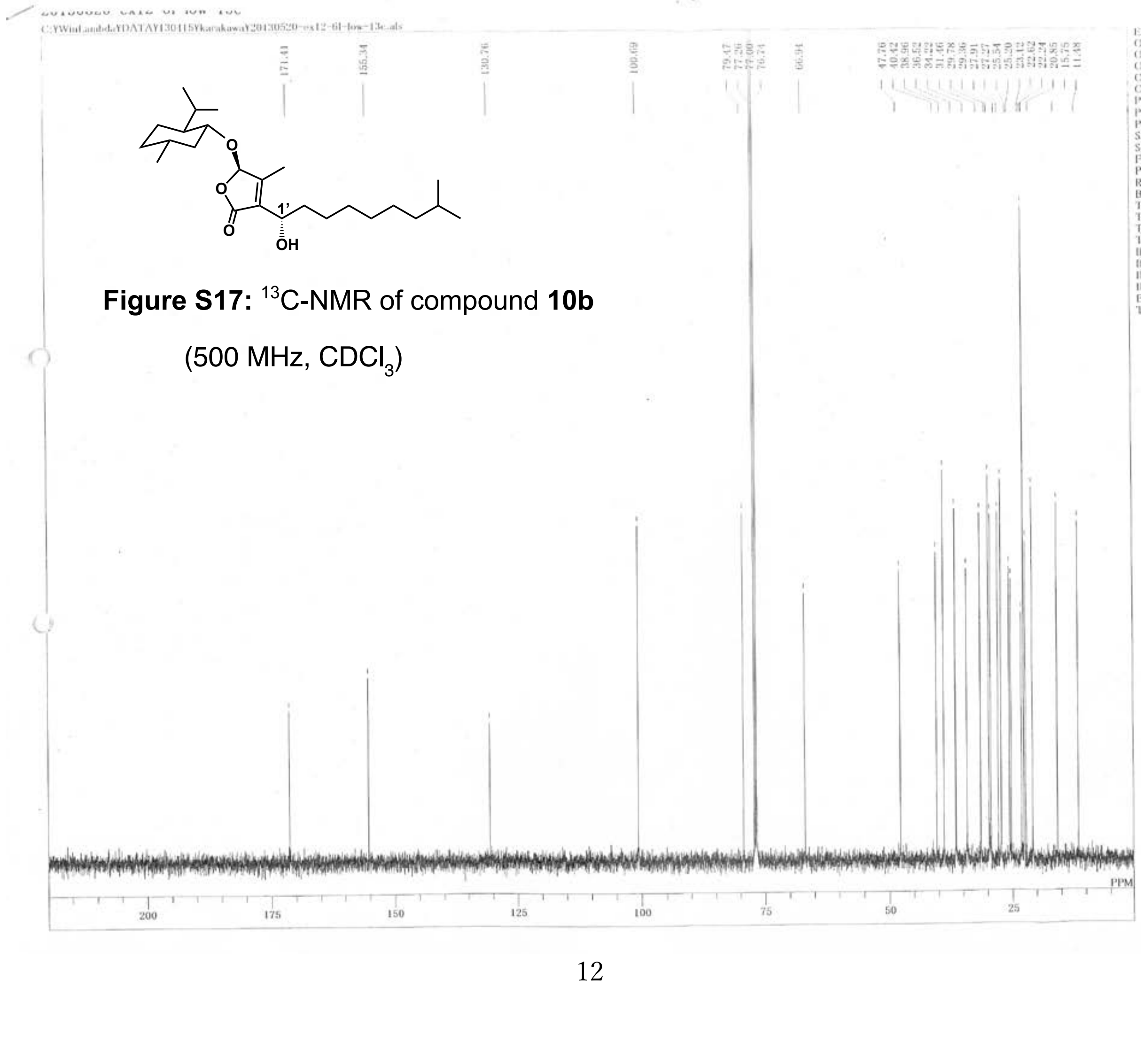

Figure S17: ^{13}C-NMR of compound **10b**

(500 MHz, $CDCl_3$)

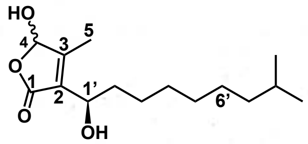

Figure S18: ^{1}H-NMR of 6'-deoxo-SRB1a (**1a**)

(500 MHz, $CDCl_3$)

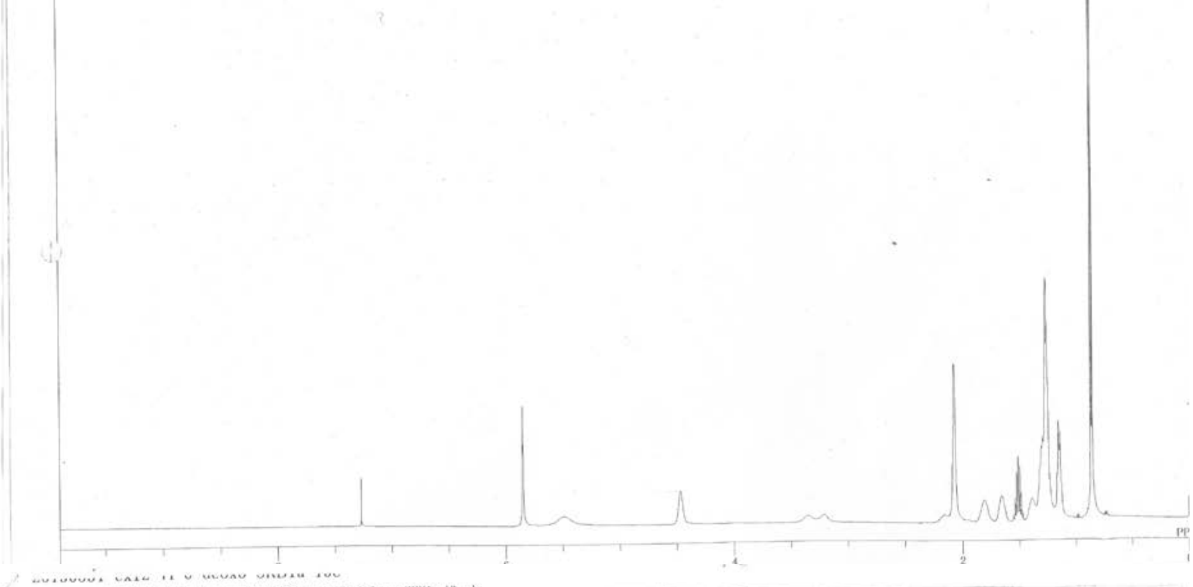

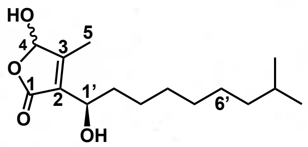

Figure S19: ^{13}C-NMR of 6'-deoxo-SRB1a (**1a**)

(500 MHz, $CDCl_3$)

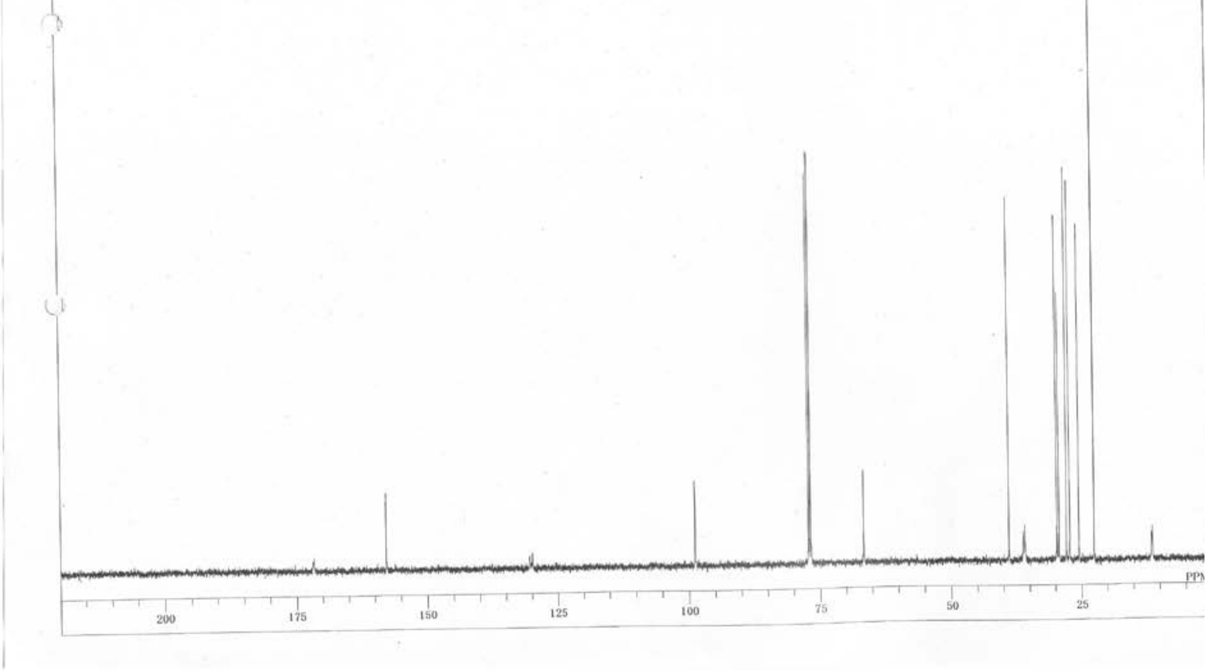

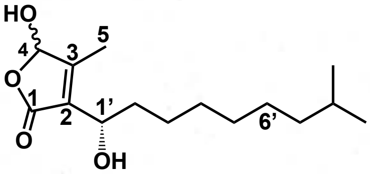

Figure S20: ^{1}H-NMR of 6’-deoxo-SRB1b (1b)

(500 MHz, $CDCl_3$)

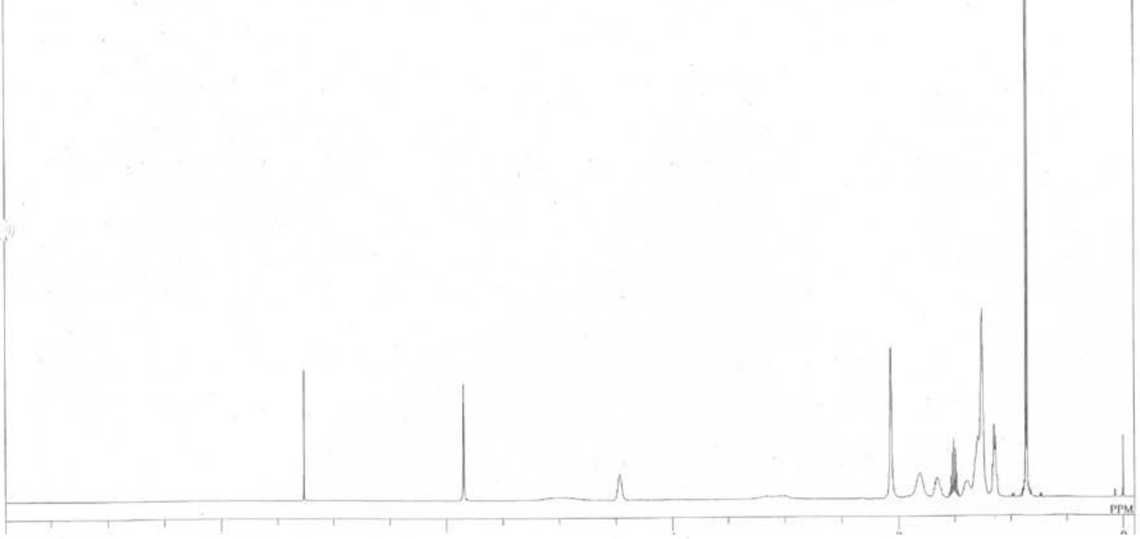

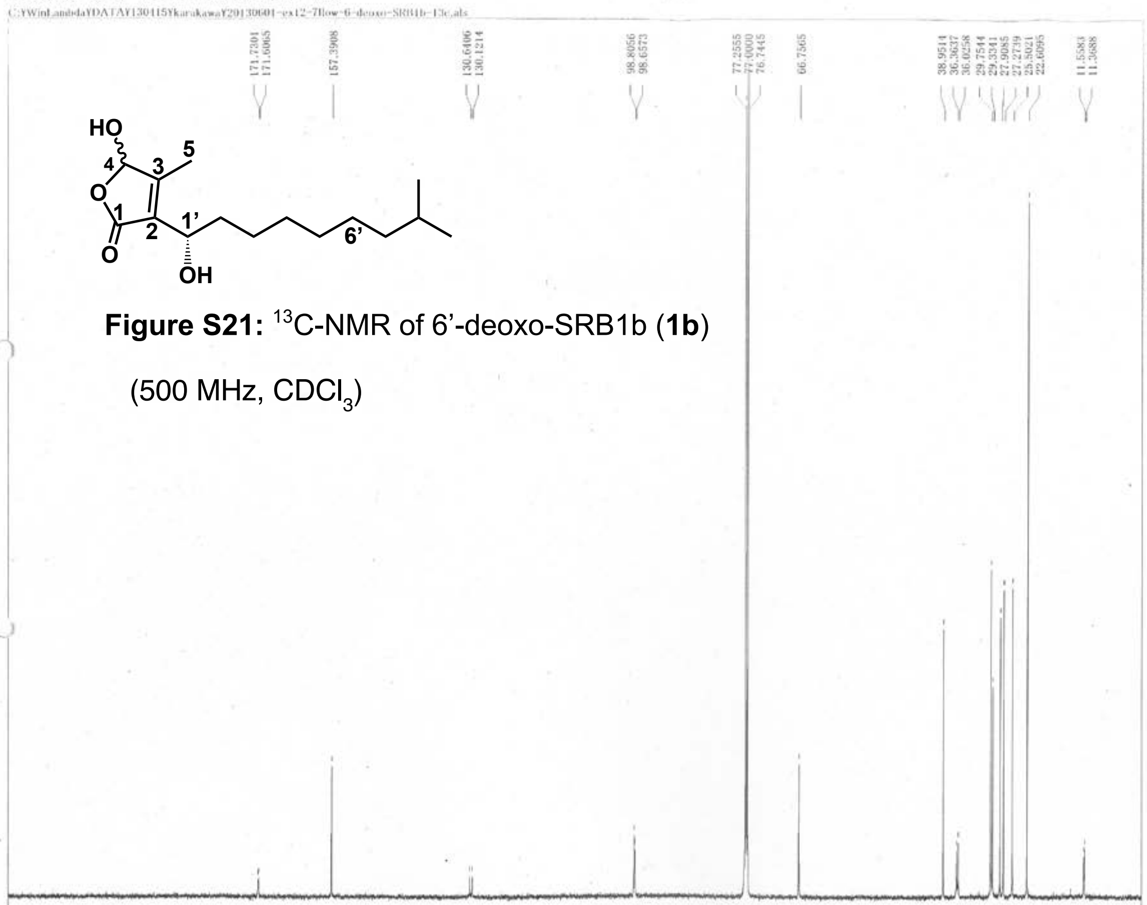

Figure S21: ^{13}C-NMR of 6’-deoxo-SRB1b (1b)

(500 MHz, $CDCl_3$)

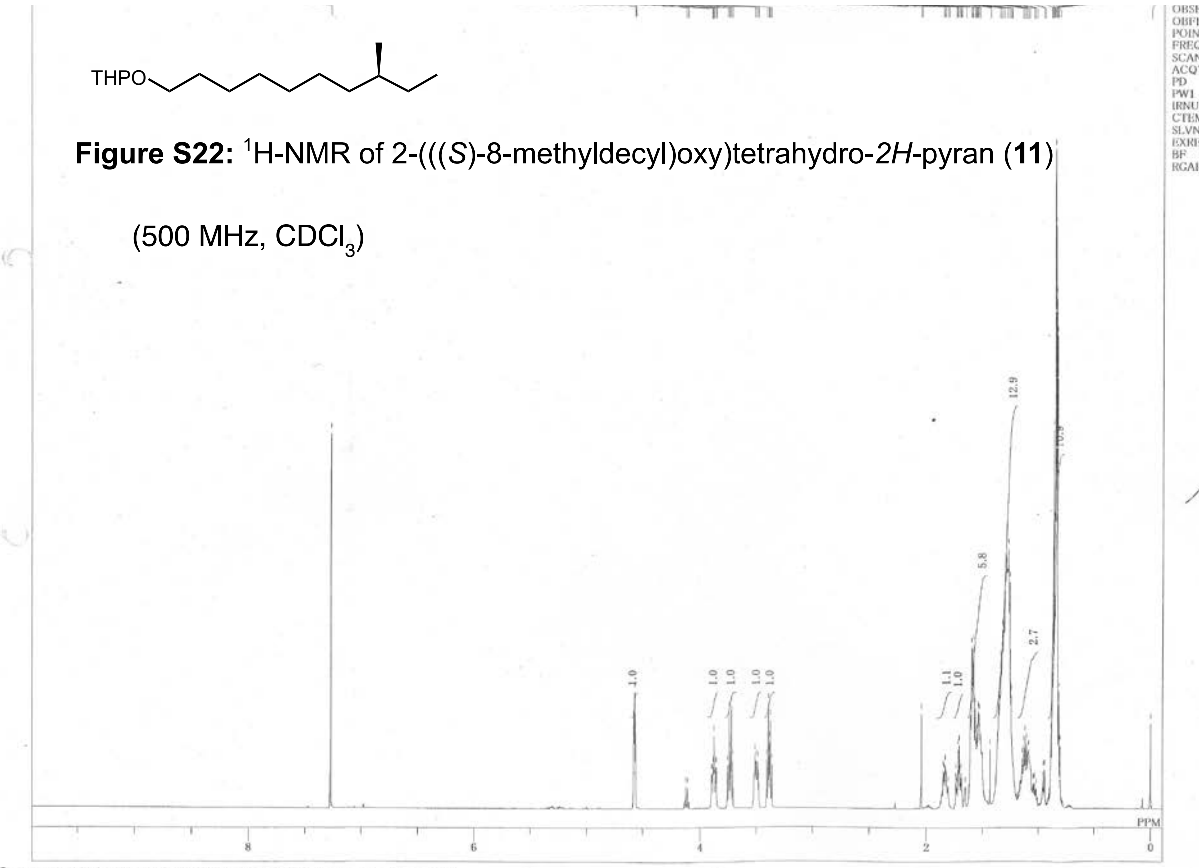

Figure S22: ^{1}H-NMR of 2-(((*S*)-8-methyldecyl)oxy)tetrahydro-*2H*-pyran (**11**)

(500 MHz, $CDCl_3$)

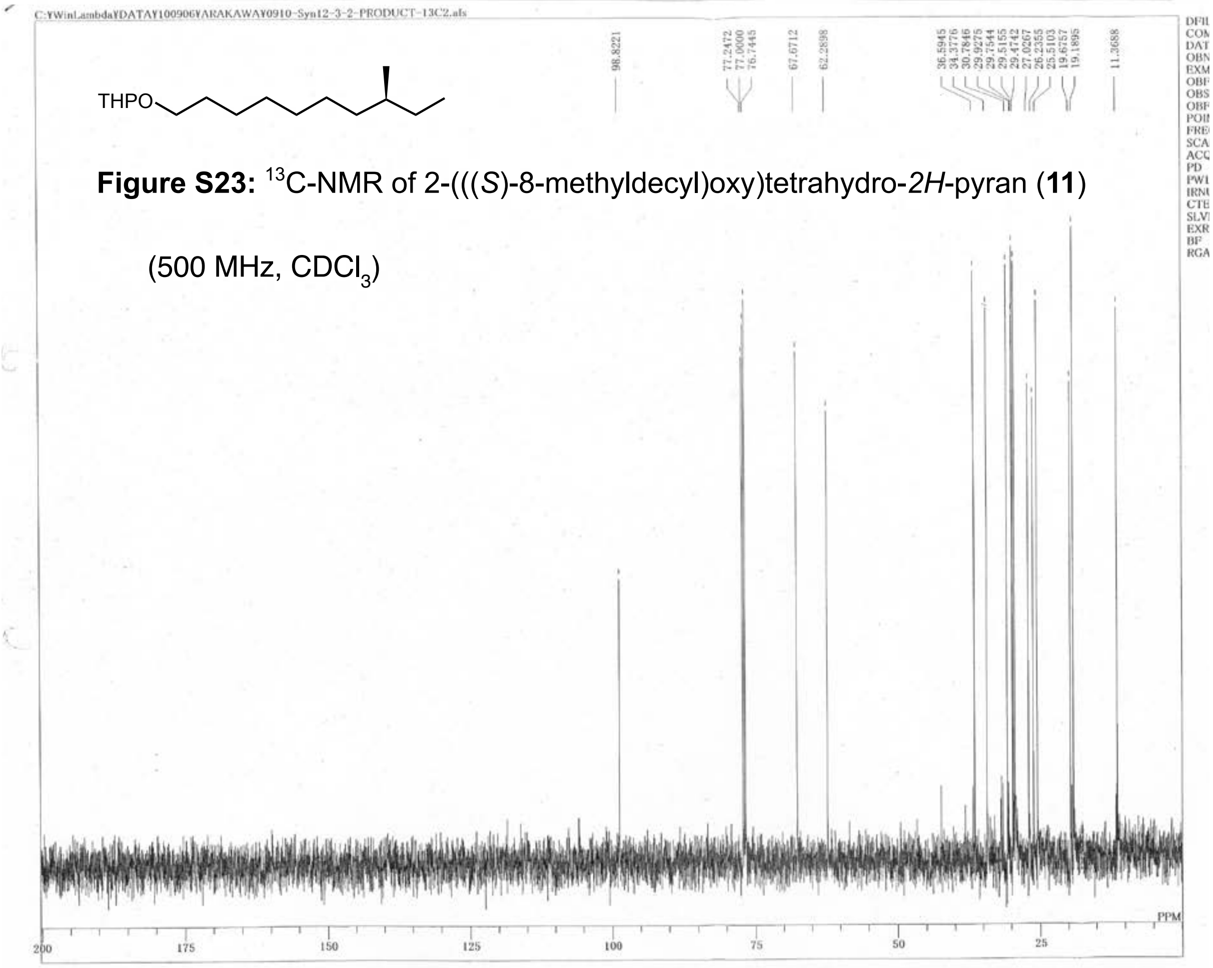

Figure S23: ^{13}C-NMR of 2-(((*S*)-8-methyldecyl)oxy)tetrahydro-*2H*-pyran (**11**)

(500 MHz, $CDCl_3$)

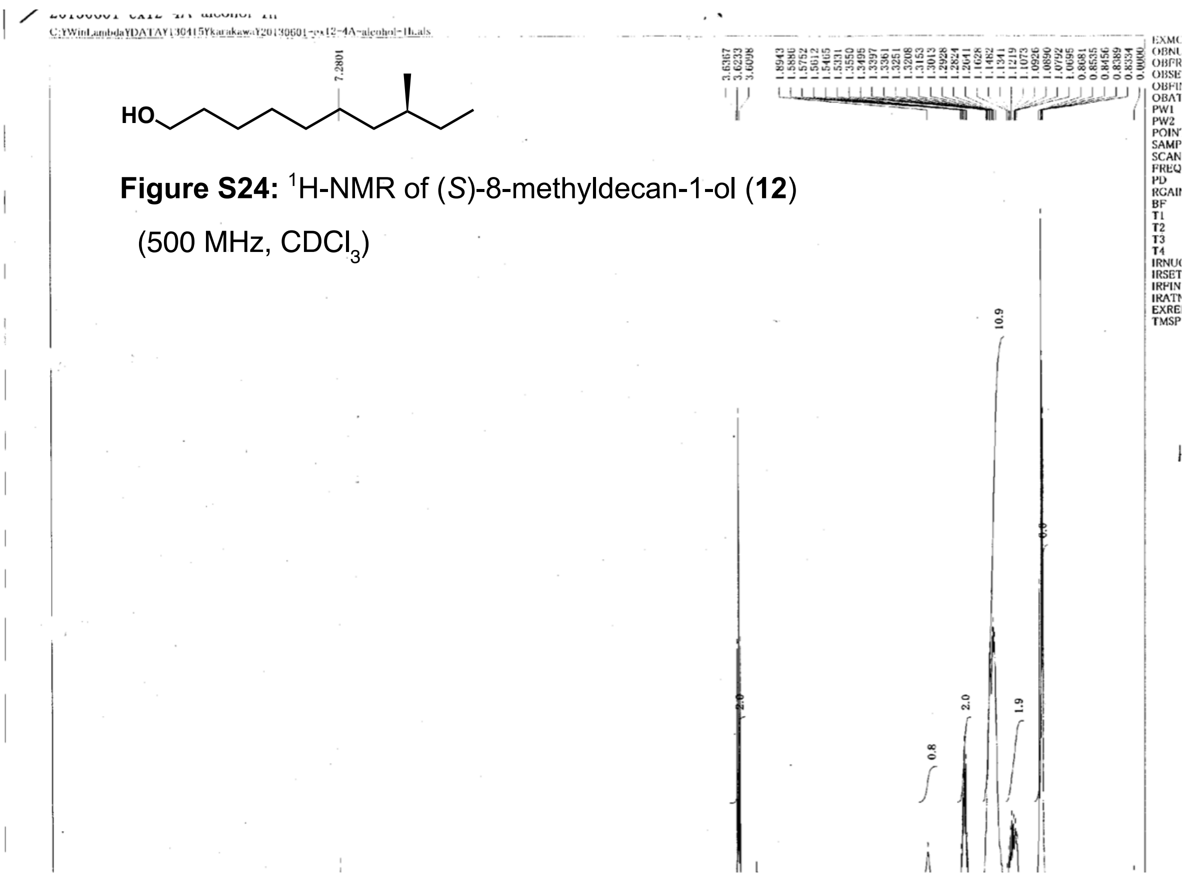

Figure S24: ^{1}H-NMR of (*S*)-8-methyldecan-1-ol (**12**)

(500 MHz, $CDCl_3$)

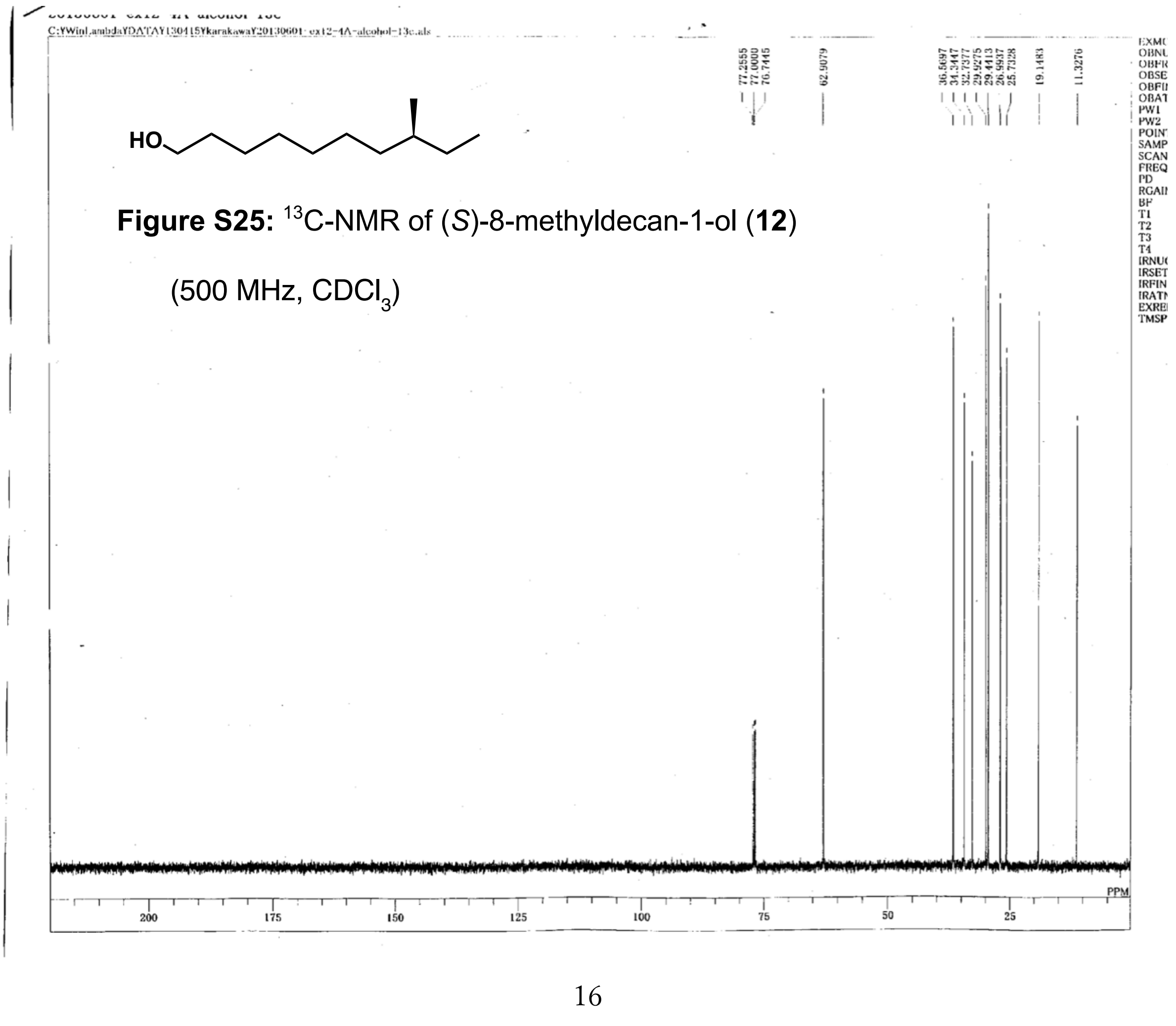

Figure S25: ^{13}C-NMR of (*S*)-8-methyldecan-1-ol (**12**)

(500 MHz, $CDCl_3$)

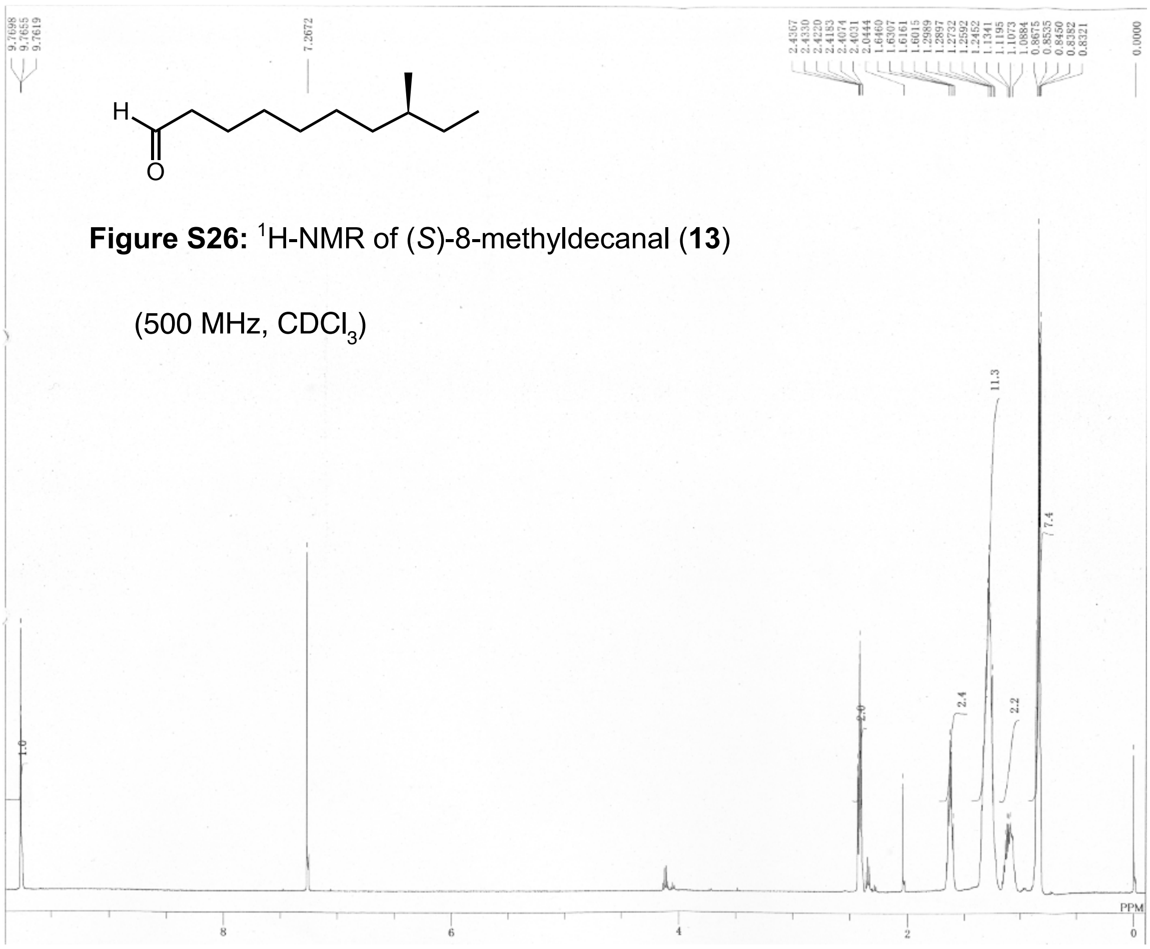

Figure S26: ^{1}H-NMR of (*S*)-8-methyldecanal (**13**)

(500 MHz, $CDCl_3$)

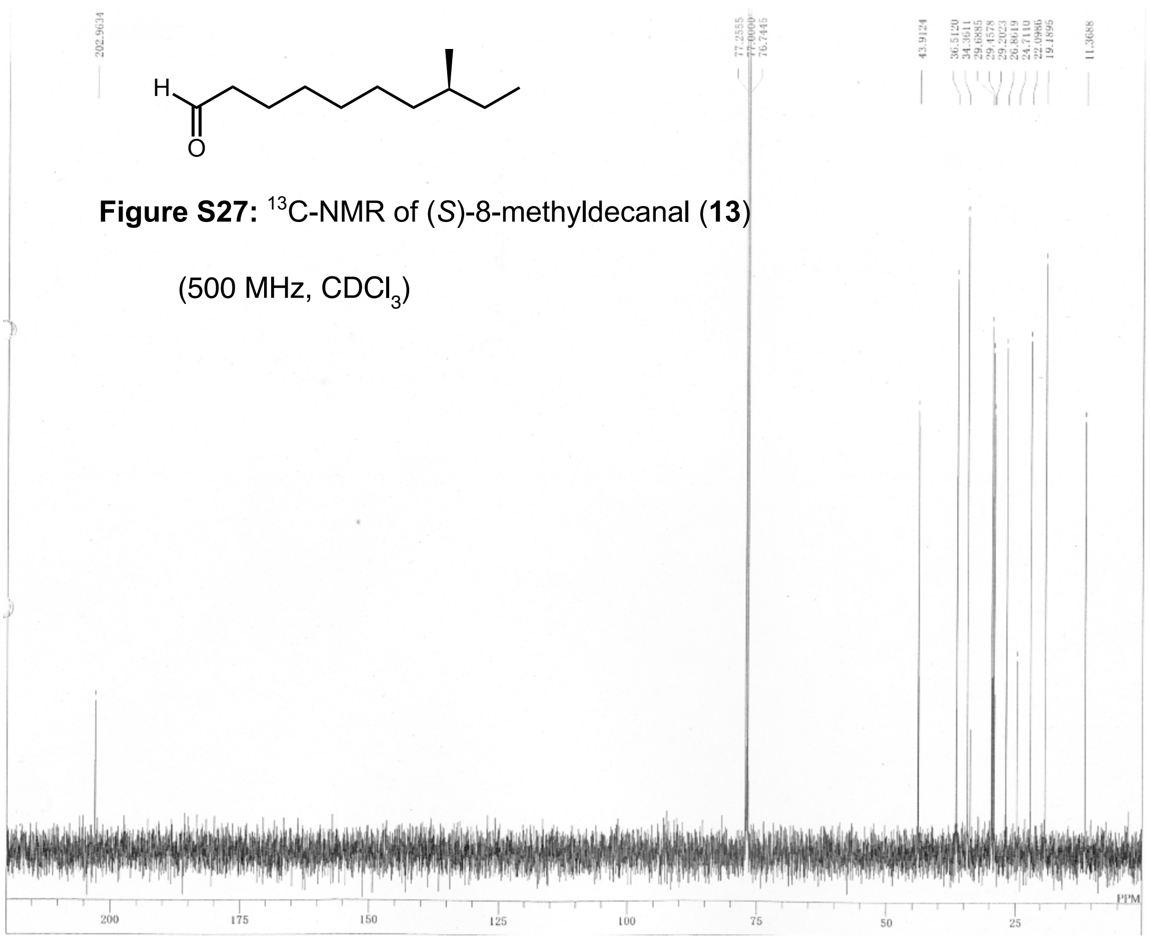

Figure S27: ^{13}C-NMR of (*S*)-8-methyldecanal (**13**)

(500 MHz, $CDCl_3$)

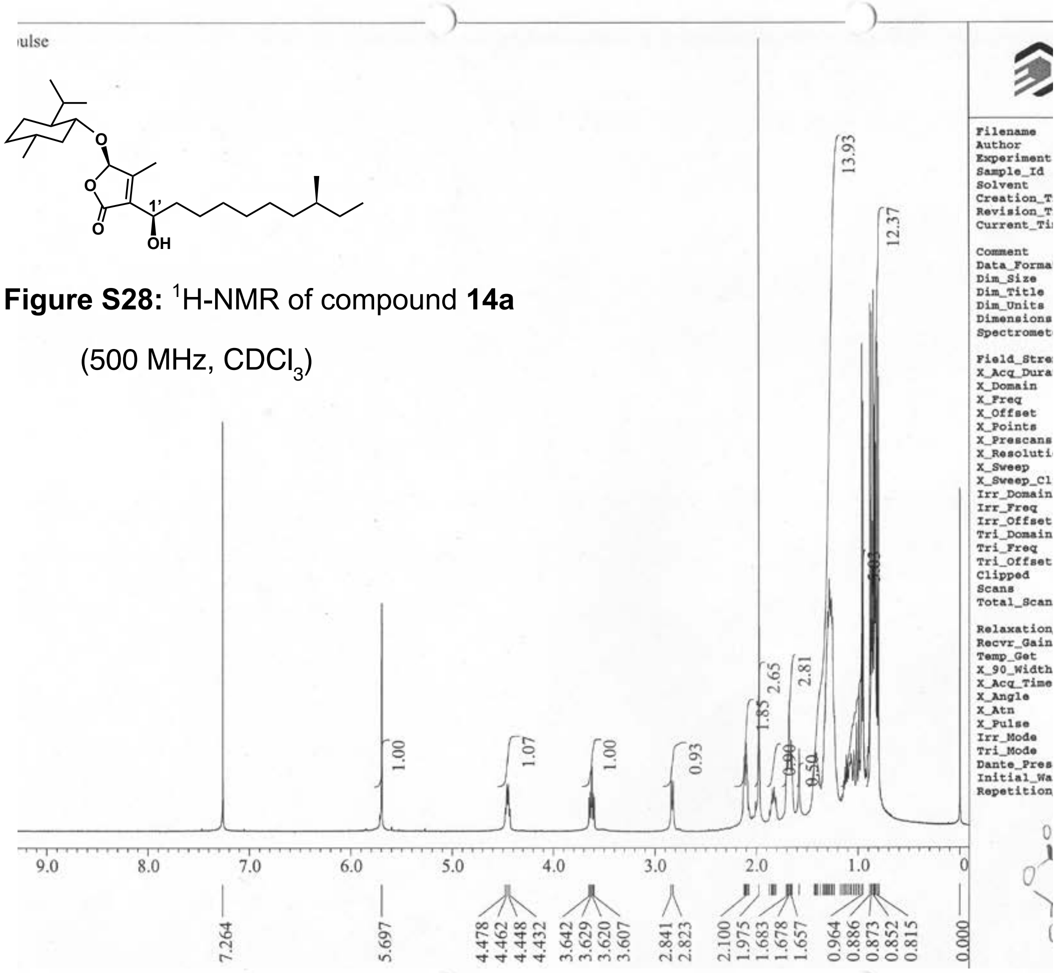

Figure S28: ^{1}H-NMR of compound **14a**

(500 MHz, $CDCl_3$)

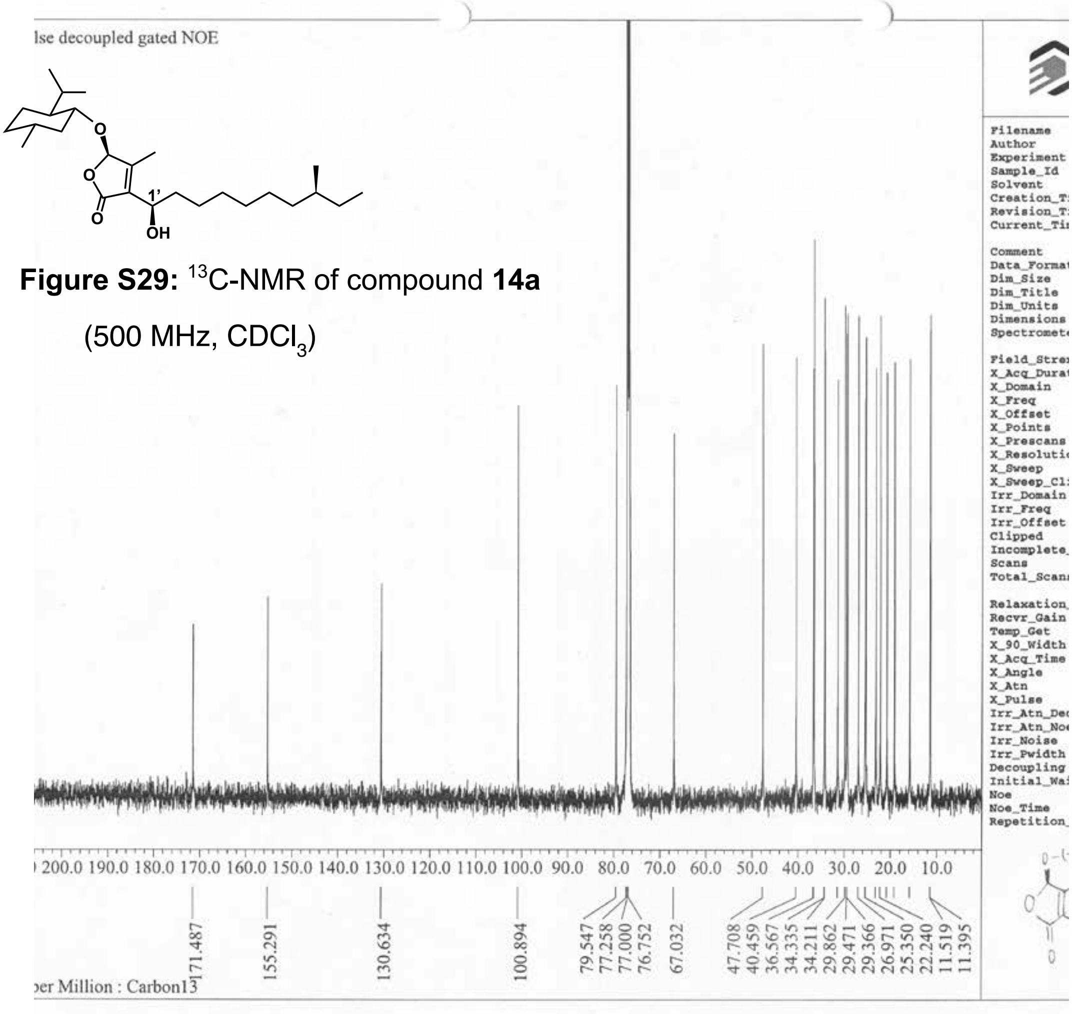

Figure S29: ^{13}C-NMR of compound **14a**

(500 MHz, $CDCl_3$)

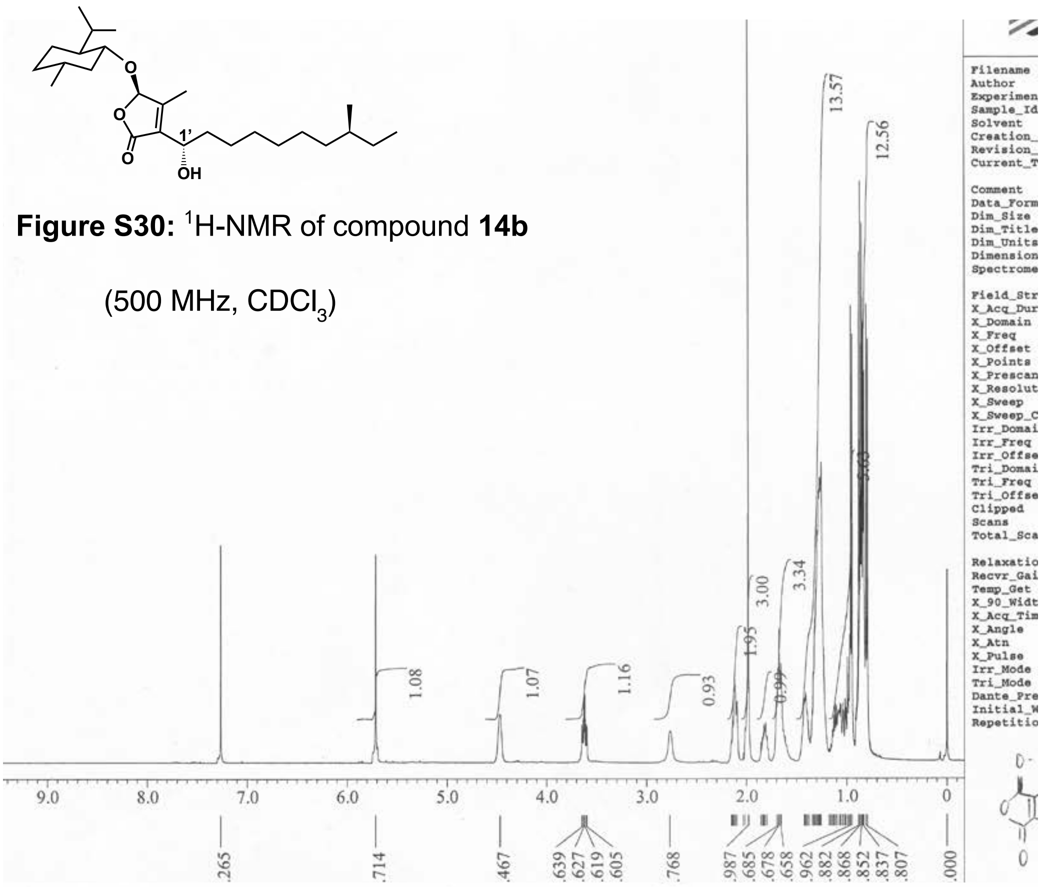

Figure S30: ^{1}H-NMR of compound **14b**

(500 MHz, $CDCl_3$)

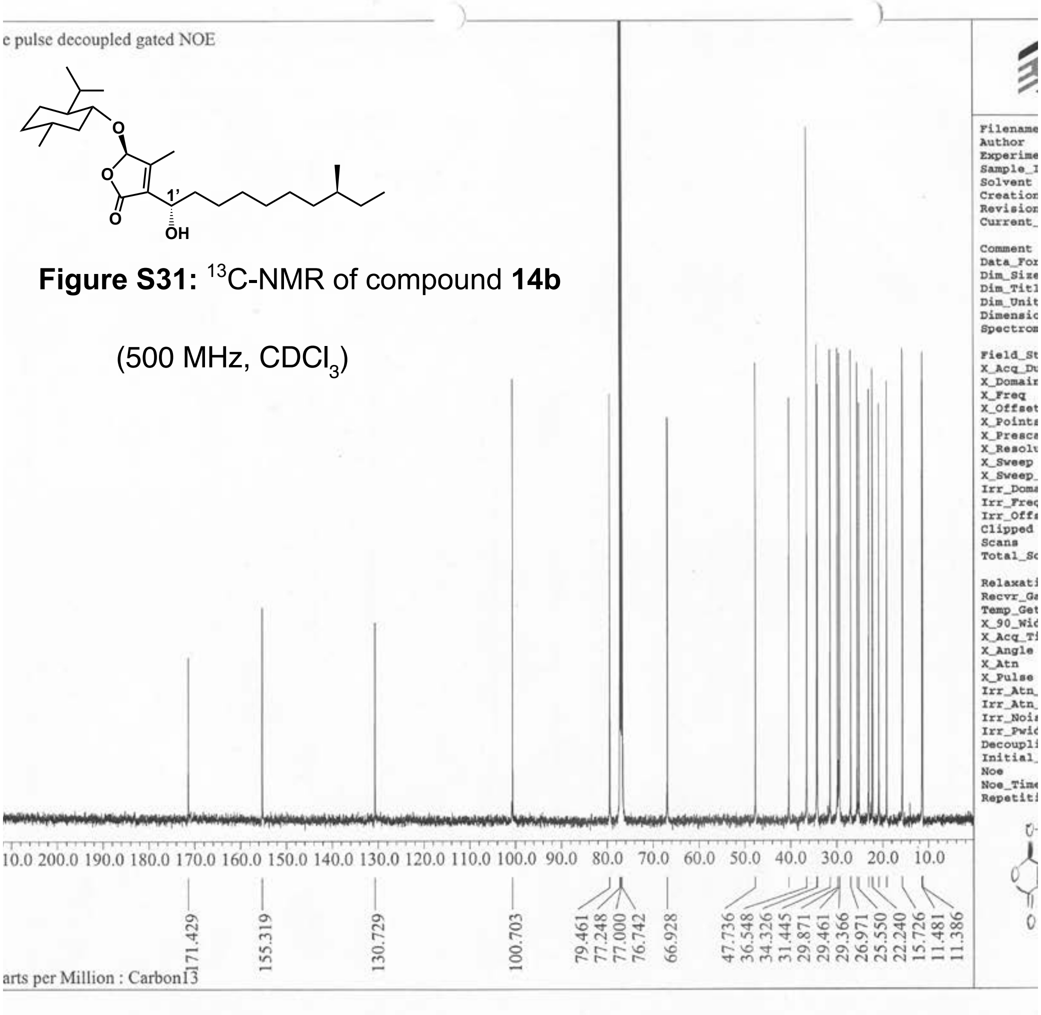

Figure S31: ^{13}C-NMR of compound **14b**

(500 MHz, $CDCl_3$)

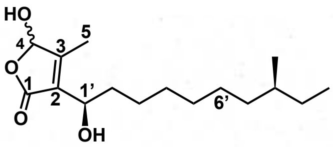

Figure S32: ^{1}H-NMR of 6'-deoxo-SRB2a (**2a**)

(500 MHz, $CDCl_3$)

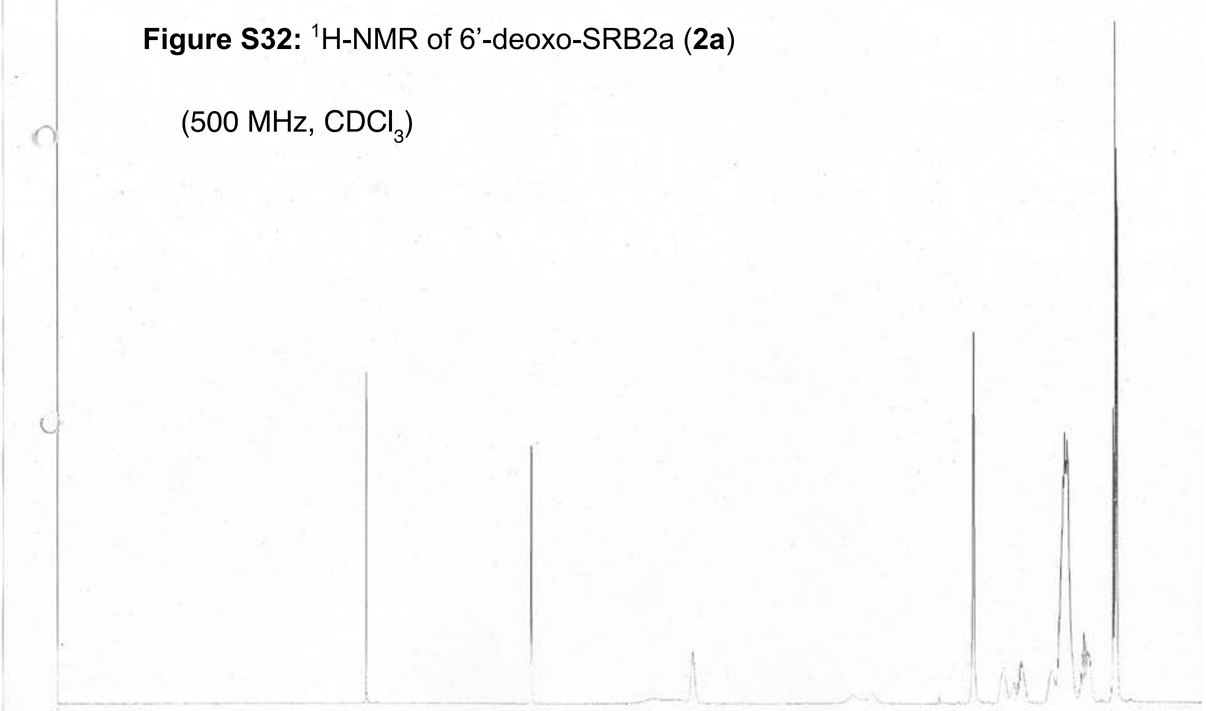

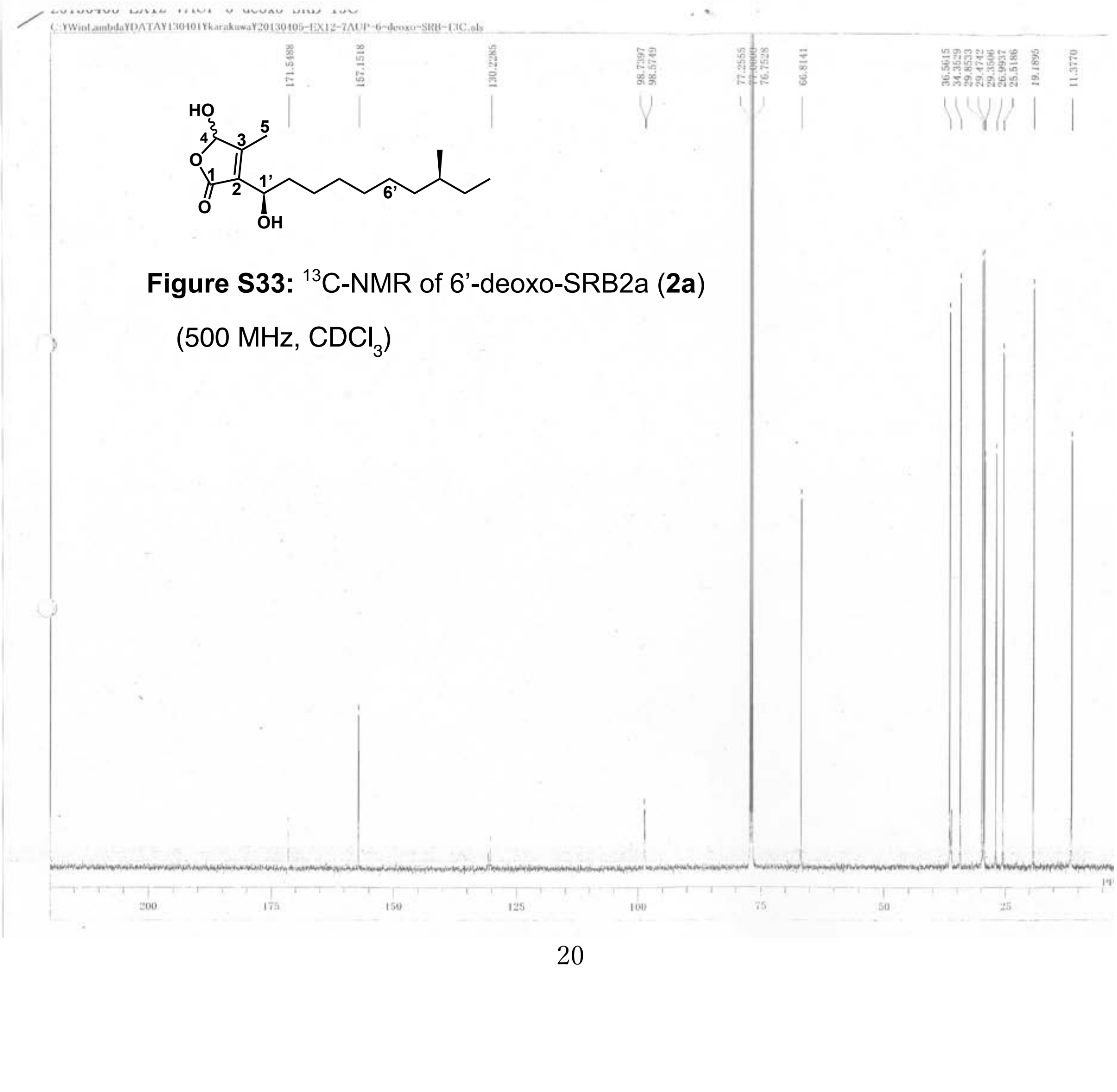

Figure S33: ^{13}C-NMR of 6'-deoxo-SRB2a (**2a**)

(500 MHz, $CDCl_3$)

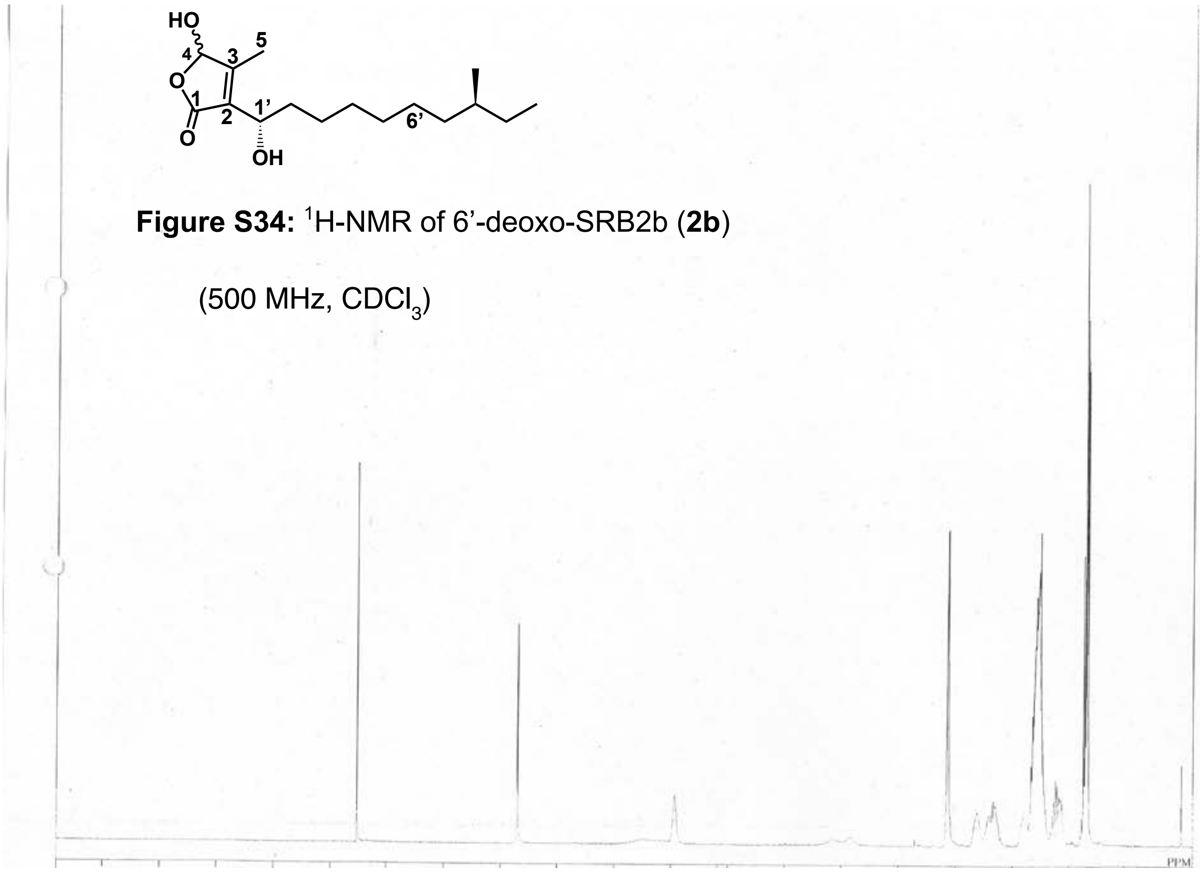

Figure S34: ^{1}H-NMR of 6'-deoxo-SRB2b (**2b**)

(500 MHz, $CDCl_3$)

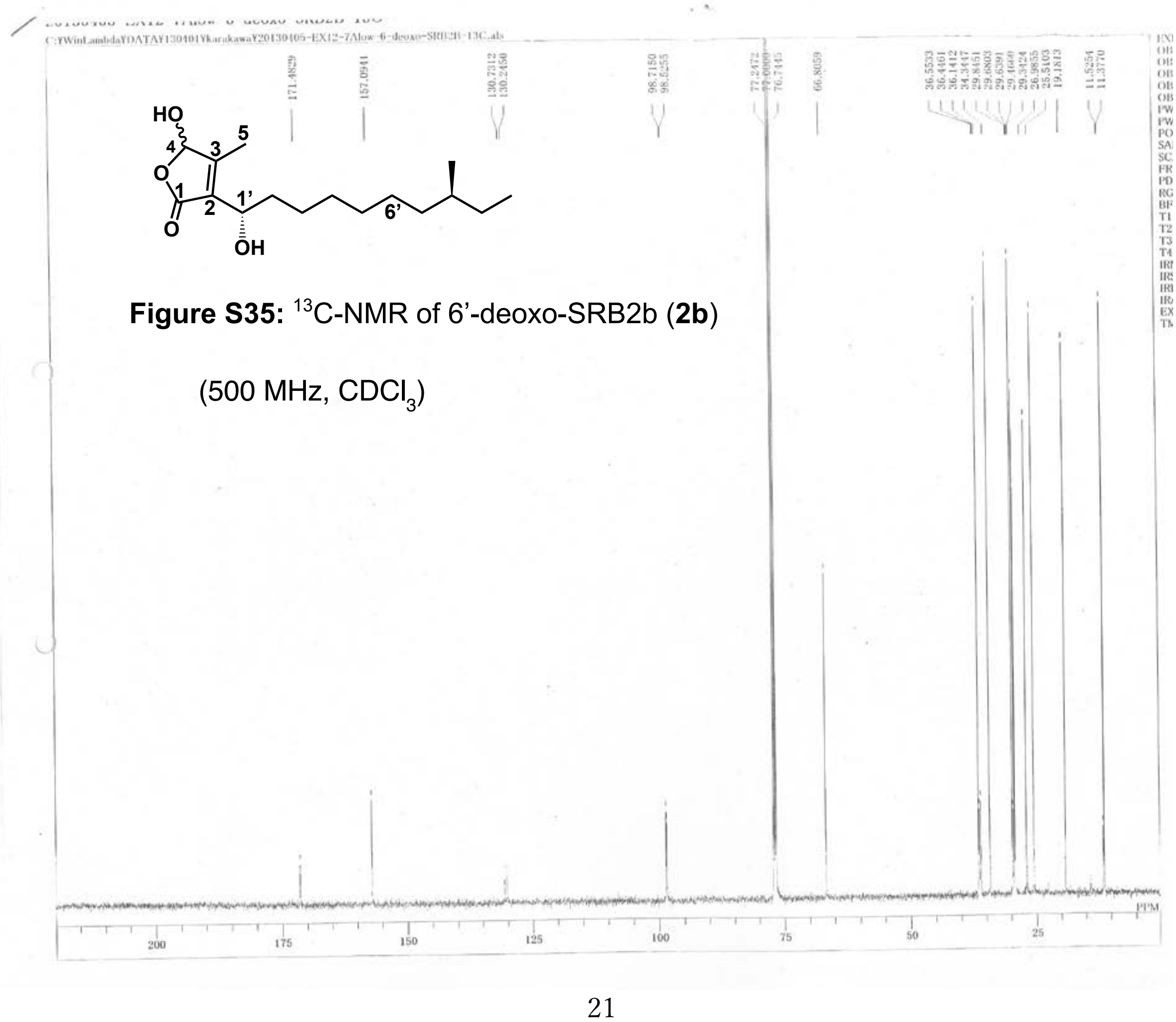

Figure S35: ^{13}C-NMR of 6'-deoxo-SRB2b (**2b**)

(500 MHz, $CDCl_3$)
